# Design ride control system using two stern flaps based 3 DOF motion modeling for wave piercing catamarans with beam seas

**DOI:** 10.1371/journal.pone.0214400

**Published:** 2019-03-25

**Authors:** Lihua Liang, Jia Yuan, Songtao Zhang, Hongyu Shi, Yanwen Liu, Peng Zhao

**Affiliations:** College of Automation, Harbin Engineering University, Harbin City, Heilongjiang Province, China; Dalian Maritime University, CHINA

## Abstract

A ride control system (RCS) based linear quadratic regulator (LQR) and genetic algorithm (GA) design is presented, to reduce the heave, roll and pitch motion (three degrees of freedom motion (3 DOF motion)) of the wave piercing catamarans (WPC) in beam waves. A detailed 3 DOF ride control model which consists of the coupling and decoupling relationships between longitudinal and transverse motion is proposed for the WPC vessel. And the complex hydrodynamic coefficients and disturbances induced by beam waves are analyzed. Moreover, two stern flaps are designed for the system in the way of alternate flapping. In the controller design, the LQR method based on GA method is adopted to reduce the 3 DOF motion of the ship. Depending on the robust search mechanism and global optimum of GA, weighting parameters can be obtained to calculate the desired gain. Finally, the motion reduction and motion sickness incidence (MSI) results demonstrate the feasibility and effectiveness of the proposed controller, and the comfort of passengers and crews can also be improved.

## 1 Introduction

Various maritime transport lines, exploitation of ocean resources and military utility could all be satisfied due to the development of the high-performance ships. Moreover, the rapid response capability and the precision strike capability in high sea states are required in modern sea warfare. In other words, the excellent seakeeping performance for modern ships is required [1]. High-speed vessels, especially wave piercing catamarans (WPC) have drawn extensive attention for their good performances. Due to the structure characteristics of the wave piercing bow and two narrow long demi-hulls of WPC vessels, the wave resistance of the ship is reduced on the basis of high speed navigation [2].

However, six degrees of freedom (6 DOF) motion (i.e. surge, sway, heave, roll, pitch and yaw) of a ship induced by sea waves can severely affect the seakeeping performance and cause the threat of comfort and safety for passengers, crews and cargos [3, 4]. To ensure the navigation safety of WPC vessels, various of stabilizers associated with the ships’ ride control system (RCS) have been developed to reduce the 6 DOF motion. Among them, T-foils, stern flaps and fins may be the most widely applied stabilizers in WPC motion control. Therefore, many researchers have devoted to the study of RCS with suitable stabilizers for WPC vessels.

“Condor 9” was built in the UK in 1990. In 1991, four fins in the bow and two fins in the amidships were added to control the pitch and roll motion. These stabilizers could withstand greater attack power and both the pitch and roll could be decreased by 15% in terrible ocean environment. However, there were some problems in the above RCS containing six fins, such as undesired sounds and lift losses etc. Hence, British researches and Australian INCAT company jointly built a ship called “Condor 10” WPC vessel. There was a T-foil installed in the bow and two flaps installed in the stern and they applied MDI company’s RCS to improve the seakeeping performance. Therefore, it is believed that ship motion could be controlled well with a suitable stabilizer and a well-designed RCS. However, it is difficult to design a control strategy for a ship model with uncertain parameters.

In order to solve the above problems, different control strategies were adopted by previous researchers for different ride control systems. The ride control of high speed WPC vessel had been studied by Australian scholars over the past few years. Haywood et al. [5] made a series of sea experiments for two catamarans with different lengths, and obtained the heave and pitch motion response curves. Davis et al. [6] recorded the heave, roll and pitch motion responses of an 86m high speed catamaran vessel, and they concluded that the system based on a T-foil and two stern tabs has good anti-heave, roll and pitch effect. Yoshida et al. [7] from Japan presented a PD control for the Resonance-Free SWATH RCS with fin stabilizers. Compared with some hull forms, such as mono-hull, ordinary SWATH and trimaran, the heave and pitch motion responses of RFS were significantly smaller in regular head waves. Our research team has been working on the integrate control system (ICS) for high-speed ships, especially for catamarans. Liang et al. [2, 8, 9] presented the two degrees of freedom (2 DOF) motion (heave and pitch) model of WPC vessel and argued the RCS for the ship based on *H*_∞_, model predictive and linear quadratic regulator (LQR) control strategies respectively.

The WPC’s ride control system based on a certain control algorithm and the different motion responses obtained from sea tests have been studied in previous researches. In the past, researches on WPC’s ride control system focused on reducing the two degrees of freedom of heave and pitch. The three degrees of freedom (3 DOF) model for the WPC vessel with the coupling relationship between longitudinal and transverse motion has not been expressed in detail in a particular sea environment. Nevertheless, the motion sickness incidence (MSI) for multiple degrees of freedom (M DOF) ride control model has not been built yet. Hence, it is important to select and determine the ride control model of multiple degrees of freedom for a ship, and then, the ship’s RCS based on a proper algorithm can be built better. However, it is difficult to build the control model of 6 DOF, because the complex and changeable marine environment causes the uncertainty of ship motion parameters [10]. Hence, according to the WPC’s structure characteristics mentioned above, only the 3 DOF motion (heave, roll and pitch motion) induced by beam seas are studied in this paper. That is to say, the longitudinal motion (heave and pitch) and the transverse motion (roll) need to be reduced at the same time.

In this paper the above mentioned weaknesses are addressed. A certain WPC RCS based on LQR-genetic algorithm (GA) is built to solve the motion control problem for a 3 DOF model in beam seas. We present a detailed 3 DOF model which consists of the coupling and decoupling relationships between longitudinal and transverse motion. Two stern flap stabilizers as the actuator of the WPC RCS are used to control the ship’s longitudinal and transverse motion synthetically with special flapping way. Major difficulties in this work include the uncertainties of hydrodynamic coefficients and the trade-off between the longitudinal and transverse motion owing to the multiple input multiple output (MIMO) system of the WPC RCS. And our research lays the foundation for the further study on the M DOF ship motion. LQR [11] is an optimal control strategy with the quadratic performance index and has been widely used in many practical systems in the past few decades, such as guided missile, spacecraft and naval vessels etc. Le et al. [12] presented a novel heading observer for 2 DOF (roll and pitch) oscillation compensation of underwater remotely operated vehicles. Xiong et al. [13] referred to the 2 DOF inverted pendulum, and presented the multi-objective optimal design with LQR method. The choices of weighting matrices (Q and R) are the main point of the LQR problem [14]. Conventionally, the weighting matrices optimization method, such as the trail and error method [15] not only consumes long time to find the global optimum solution but also increases the difficulty of practical project realization. Hence, the GA method, inspired by Holland [16], is designed to optimize the important parameters of some proposed controllers. Depending on its self-adapting global optimization search and the operation of the natural selection mechanism, GA is the evolutionary computations by computer simulation. Chang et al. [17] proposed sliding controller, which employs the applicable GA to reduce the influence of mismatched disturbance. Finally, the results of a numeric example demonstrated the effectiveness of the developed controller. Kumarawadu et al. [18] presented the neural networks-based adaptive control using GA method to enhance the output tracking performance of partly known robotic systems. And GA is used to find the best combination of switching function. Since the ship’s ride control system is the multiple input multiple output system, in which the inputs are two flaps’ attack angles, and the outputs are the ship motion state variables (i.e. heave, roll and pitch), LQR control strategy is considered to control the WPC motion. And due to the powerful search mechanisms of GA, it can optimize the parameters Q and R in LQR controller. The main contribution of this paper is to ensure that the RCS with two stern flaps based on the proposed controller is feasible and effective for the M DOF motion of WPC vessels in beam seas.

The structure of this paper is organized as follows. Section 2 describes the mathematical models of 6 DOF for ships and 3 DOF motion for the WPC vessels, in which the hydrodynamic coefficients, ocean waves and the actuators of 3 DOF model are elaborated. Furthermore, the ride control system with two stern flaps for the WPC is built. Section 3 presents the design of linear quadratic regulator controller based on genetic algorithm. In Section 4, results of software simulation are described and motion sickness incidence is proposed to verify the feasibility and effectiveness of the proposed control approach. Finally, conclusions are made in Section 5.

## 2 Mathematical model

### 2.1 The six degrees of freedom model of a ship

**Fig 1:**
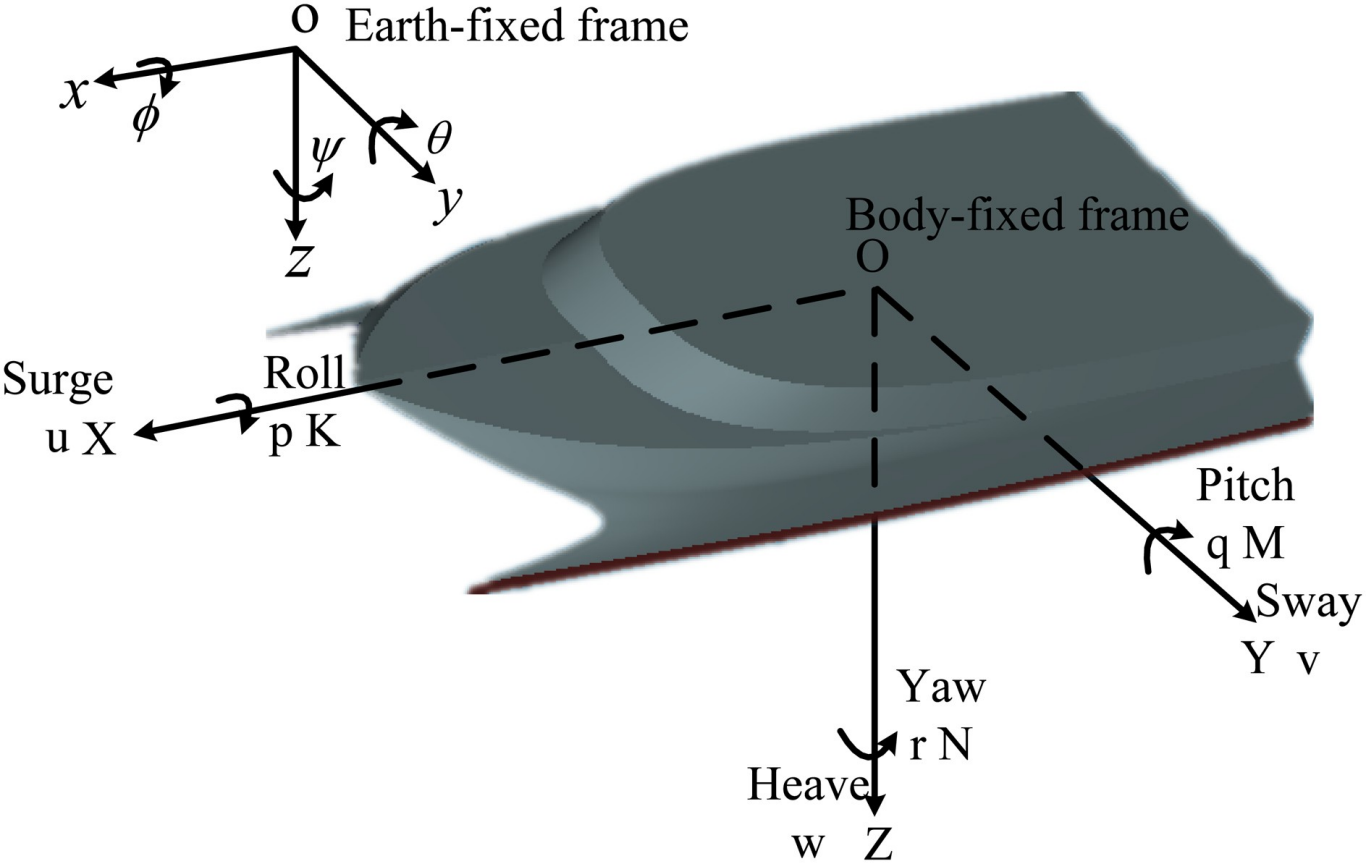
Standard notation and sign conventions for a ship motion description.

When a ship navigation is not limited to the head seas, there is 6 DOF motion for a ship by irregular waves from different directions in bad weather conditions. Fig 1 shows the 6 DOF motion of a ship in the earth-fixed and body-fixed coordinate frames. And the general model structure [19] can be conveniently expressed as:

$$\begin{array}{c} M\dot{\upsilon }+D\left(\upsilon \right)\upsilon +g\left(\eta \right)=\tau_{waves}+\tau_{control} \end{array}$$ (1)

Where $\dot{\upsilon }$ denotes the translational and rotational accelerations of a ship, *υ* denotes the translational and rotational velocities of a ship, *η* denotes the positions and orientations of a ship, *τ* denotes the forces and moments of a ship. The detailed description of the above parameters are shown in Table 1. *M* is the inertia matrix, *D*(*υ*) is the hydrodynamic damping matrix, including potential damping, skin friction, wave drift damping and damping due to vortex shedding, *g*(*η*) is the vector of gravitational force and moment, *τ*_*waves*_ is commonly described as the wave disturbance force and moment and *τ*_*control*_ is the control force and moment [20].

**Table 1:** Notations used for a ship.

DOF	Motion descriptions	Positions and orientations *η*	Translational and rotational velocities *υ*	Forces and Moments *τ*
1	Surge (motion in the *x*-direction)	*x*	*u*	*X*
2	Sway (motion in the *y*-direction)	*y*	*v*	*Y*
3	Heave (motion in the *z*-direction)	*z*	*w*	*Z*
4	Roll (Rotation about *x*-axis)	*ϕ*	*p*	*K*
5	Pitch (Rotation about *y*-axis)	*θ*	*q*	*M*
6	Yaw (Rotation about *z*-axis)	*ψ*	*r*	*N*

When the origin of the body-fixed frame is located at the center of gravity of the ship, and the coordinate axes are consistent with the axes of inertia of the ship, neglecting small and higher-order hydrodynamic coefficients, according to the coupling strength between six degrees of freedom motion, the general model formula can be expanded as follows [21, 22]:

$$\begin{array}{c} \begin{array}{cc} & \left(m-X_{\dot{u}}\right)\dot{u}-X_{u}u-X_{u|u|}u\left|u\right|-X_{\dot{v}}\dot{v}-X_{v}v-X_{\dot{w}}\dot{w}-X_{w}w\\ & -X_{\dot{p}}\dot{p}-X_{p}p-X_{\dot{q}}\dot{q}-X_{q}q-X_{q|q|}q|q|-X_{\dot{r}}\dot{r}-X_{r}r-X_{r|r|}r\left|r\right|\\ & +\left(m-X_{wq}\right)wq-\left(m+X_{vr}\right)vr-mg\sin\theta =X_{waves}+X_{control}\\ & \left(m-Y_{\dot{v}}\right)\dot{v}-Y_{v}v-Y_{v|v|}v\left|v\right|-Y_{\dot{w}}\dot{w}-Y_{w}w-Y_{\dot{p}}\dot{p}-Y_{p}p-Y_{\dot{q}}\dot{q}\\ & -Y_{q}q-Y_{\dot{r}}\dot{r}-Y_{r}r-Y_{r|r|}r\left|r\right|+\left(m-Y_{ur}\right)ur-\left(m+Y_{wp}\right)wp\\ & -Y_{uv}uv-Y_{pq}pq+mg\sin\phi \cos\theta =Y_{waves}+Y_{control}\\ & \left(m-Z_{\dot{w}}\right)\dot{w}-Z_{w}w-Z_{w|w|}w|w|-Z_{\dot{q}}\dot{q}-Z_{q}q-Z_{q|q|}q\left|q\right|-Z_{\dot{u}}\dot{u}\\ & -Z_{u}u-Z_{\dot{p}}\dot{p}-Z_{p}p-Z_{\dot{r}}\dot{r}-Z_{r}r+\left(m-Z_{vp}\right)vp-\left(m+Z_{uq}\right)uq\\ & -Z_{uw}uw-Z_{pr}pr+mg\cos\phi \cos\theta =Z_{waves}+Z_{control}\\ & \left(I_{xx}-K_{\dot{p}}\right)\dot{p}-K_{p}p-K_{p|p|}p\left|p\right|-K_{\dot{v}}\dot{v}-K_{v}v-K_{\dot{q}}\dot{q}-K_{q}q\\ & -K_{\dot{r}}\dot{r}-K_{r}r+\left(I_{zz}-I_{yy}\right)qr-\rho g\nabla \bar{GM_{T}}=K_{waves}+K_{control}\\ & \left(I_{yy}-M_{\dot{q}}\right)\dot{q}-M_{q}q-M_{q|q|}q|q|-M_{\dot{w}}\dot{w}-M_{w}w-M_{w|w|}w\left|w\right|\\ & -M_{\dot{u}}\dot{u}-M_{u}u-M_{\dot{r}}\dot{r}-M_{r}r+\left(I_{xx}-I_{zz}-M_{pr}\right)pr-M_{uw}uw\\ & -M_{uq}uq-M_{vp}vp-\rho g\nabla \bar{GM_{L}}=M_{waves}+M_{control}\\ & \left(I_{zz}-N_{\dot{r}}\right)\dot{r}-N_{r}r-N_{r|r|}r|r|-N_{\dot{v}}\dot{v}-N_{v}v-N_{v|v|}v\left|v\right|-N_{\dot{p}}\dot{p}-N_{p}p\\ & +\left(I_{yy}-I_{xx}-N_{pq}\right)pq-N_{uv}uv-N_{ur}ur-N_{wp}wp=N_{waves}+N_{control} \end{array} \end{array}$$ (2)

Where *m* is the ship mass, *g* is the acceleration of gravity, *I*_*xx*_, *I*_*yy*_ and *I*_*zz*_ are the inertias about the *X*, *Y* and *Z*-axis, respectively, *ρ* is the water density, ∇ is the ship displacement volume, $\bar{GM_{T}}$ and $\bar{GM_{L}}$ are the transverse and longitudinal metacentric heights, respectively, $X_{\dot{u}}$, *X*_*u*_, …, *N*_*wp*_ are the hydrodynamic coefficients of the ship, they are defined in this way: $X_{\dot{u}}=\frac{dX}{d\dot{u}}$, $X_{u}=\frac{dX}{du}$, …, $N_{wp}=\frac{\partial^{2}N}{\partial w\partial p}$, in which terms related to displacements or angles are the hydrostatic restoring coefficients, terms related to velocities are the hydrodynamic damping coefficients and terms related to the accelerations are the fluid inertia coefficients.

### 2.2 The three degrees of freedom model of WPC

There are two classes of motion for a ship, one is coupled motion and the other is approximate decoupled motion. Furthermore, one degree of freedom (1 DOF) motion will inevitably effect other motion in the same class [23]. However, due to the uncertainty and complexity of the external disturbances (such as winds, ocean waves, and ocean currents etc.), the 6 DOF hydrodynamic parameters of a ship are more complicated and changeable, and it will be more difficult to get the higher order coupling coefficients. In this case, we only consider the ocean waves as the main disturbance to study the main motion for a WPC vessel. According to the characteristics of WPC vessels, the heave and pitch motion are obvious, when the ship sails in head seas. When the direction of waves turns into the beam seas direction, the roll motion becomes severe, especially for encounter angle of 90deg [24, 25]. Therefore, heave, roll and pitch are studied in this paper under beam seas. The heave and pitch are coupled with each other, and they are uncoupled with roll, illumined by [10]. According to Eq (2), the nonlinear equations in 3 DOF motion based on the stern flap stabilizer model are described as following:

$$\begin{array}{c} \begin{array}{cc} & \left(m-Z_{\dot{w}}\right)\dot{w}-Z_{w}w-Z_{w|w|}w|w|-Z_{\dot{q}}\dot{q}-Z_{q}q-Z_{q|q|}q\left|q\right|+mg\cos\phi \cos\theta \\ & =Z_{waves}+Z_{flap}\\ & \left(I_{xx}-K_{\dot{p}}\right)\dot{p}-K_{p}p-K_{p|p|}p\left|p\right|-\rho g\nabla \bar{GM_{T}}=K_{waves}+K_{flap}\\ & \left(I_{yy}-M_{\dot{q}}\right)\dot{q}-M_{q}q-M_{q|q|}q|q|-M_{\dot{w}}\dot{w}-M_{w}w-M_{w|w|}w\left|w\right|-\rho g\nabla \bar{GM_{L}}\\ & =M_{waves}+M_{flap} \end{array} \end{array}$$ (3)

Where *Z*_*flap*_ are the forces in the *Z* direction. *K*_*flap*_ and *M*_*flap*_ are the moments with respect to the *X* and *Y* axes, respectively. And they are provided by the anti-heave, roll and pitch actuators-stern flaps.

#### 2.2.1 Hydrodynamic parameters of 3 DOF

The calculation of hydrodynamic parameters in Eq (3) is a difficult point in our study, and is also the key point to design the controller for the WPC RCS. The hydrodynamic parameters are complicated and changeable in different sea states, speeds and wave encounter angles. At present, the hydrodynamic parameters can be obtained by three ways.

1. Formula estimation method.
2. Software simulation method.
3. Towing tank test method.

**Fig 2:**
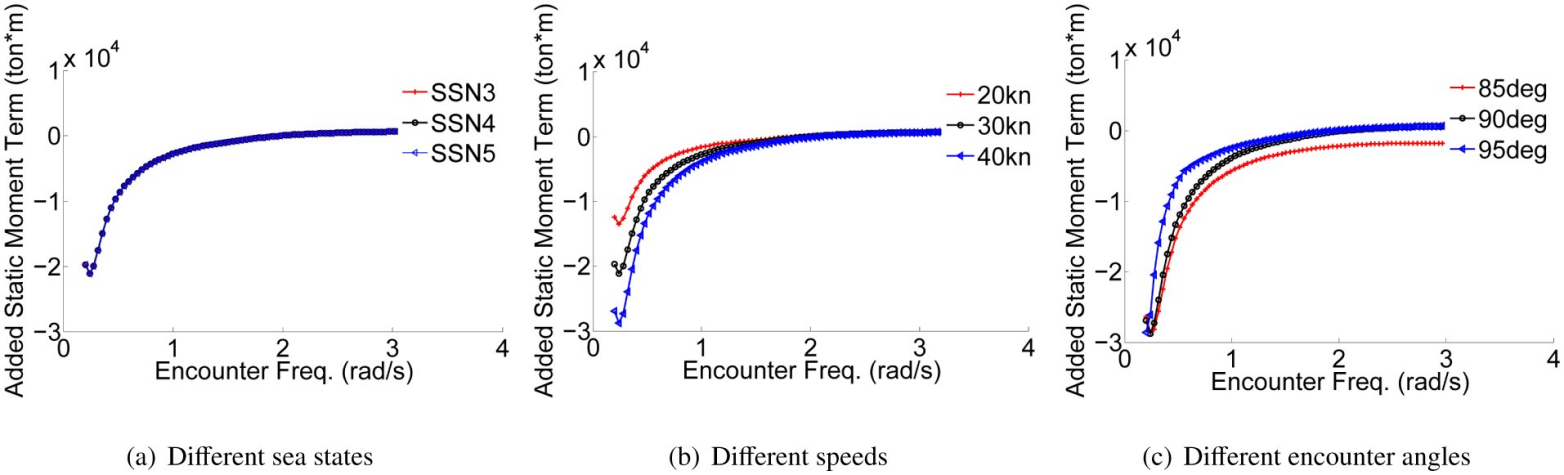
The longitudinal hydrodynamic parameters in different situations. (a) Different sea states. (b) Different speeds. (c) Different encounter angles.

**Fig 3:**
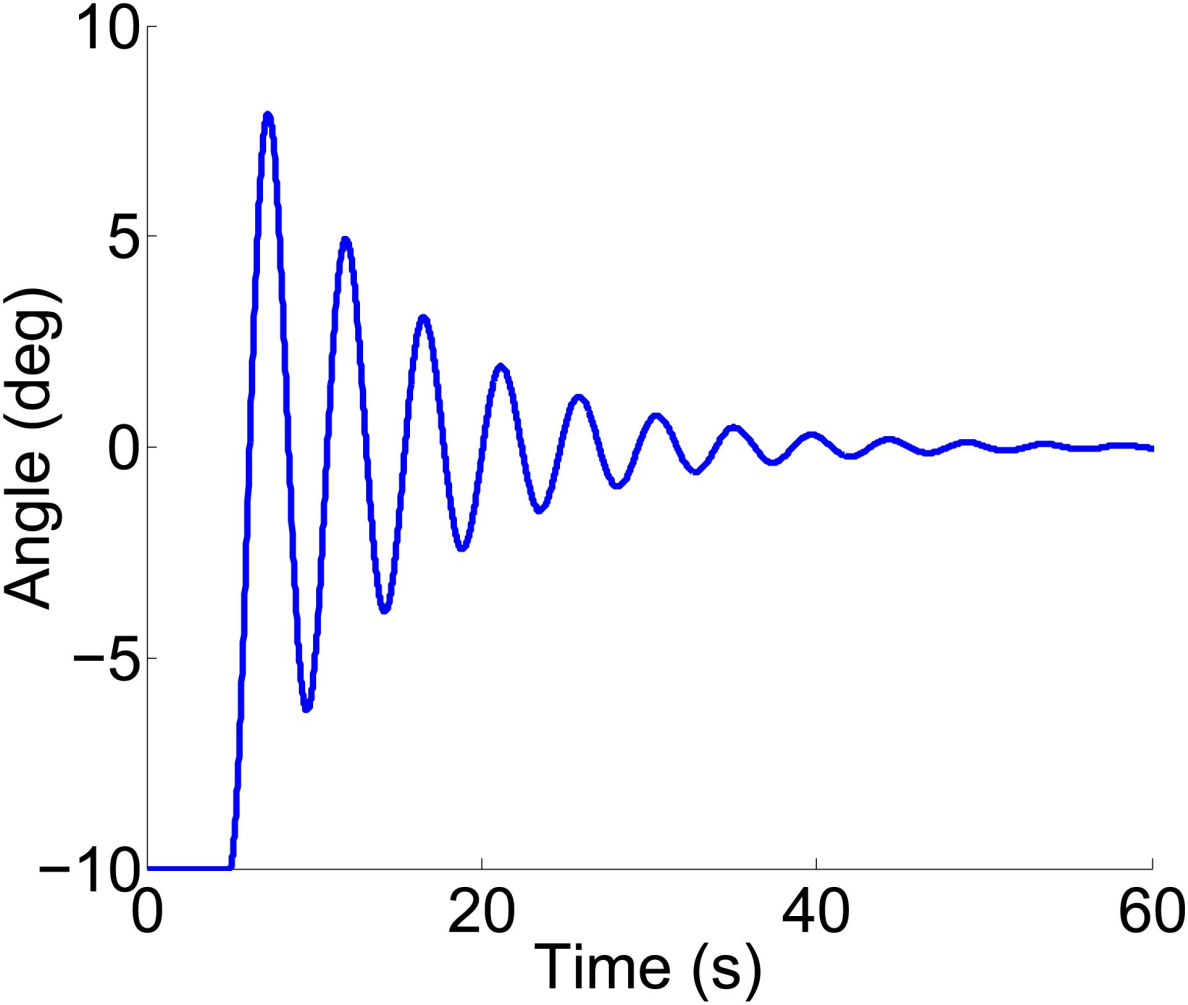
Free roll motion of the WPC vessel.

*A*. *Longitudinal hydrodynamic parameters analysis*.
  The coefficients of 2 DOF motion (heave and pitch) are solved using the the computer aided tool for a 90m WPC vessel, and the principal scale parameters of the hull can be found in [2]. Fig 2 shows the comparison results of a certain series of coefficients in different situations. It can be seen that the longitudinal coefficient is almost no fluctuations for changes in the sea state numbers (SSN). At low frequency, the coefficient decreases with the increase of speed. However, with the increase of encounter angle, the coefficient will increase. At high frequency, these coefficient values will eventually tend to be consistent.
*B*. *Roll hydrodynamic parameters analysis*.
  Software simulation method is combined with the formula estimation method to solve the roll coefficients in this paper. It can be seen that the coefficients are estimated using the free roll motion of the WPC vessel as shown in Fig 3. Illumined by [26], the moment of inertia $I_{xx}-K_{\dot{p}}$, the hydrodynamic damping term *K*_*p*_ and the coefficients of restoring force $\rho g\nabla \bar{GM_{L}}$ can be obtained.

#### 2.2.2 Wave disturbances

**Fig 4:**
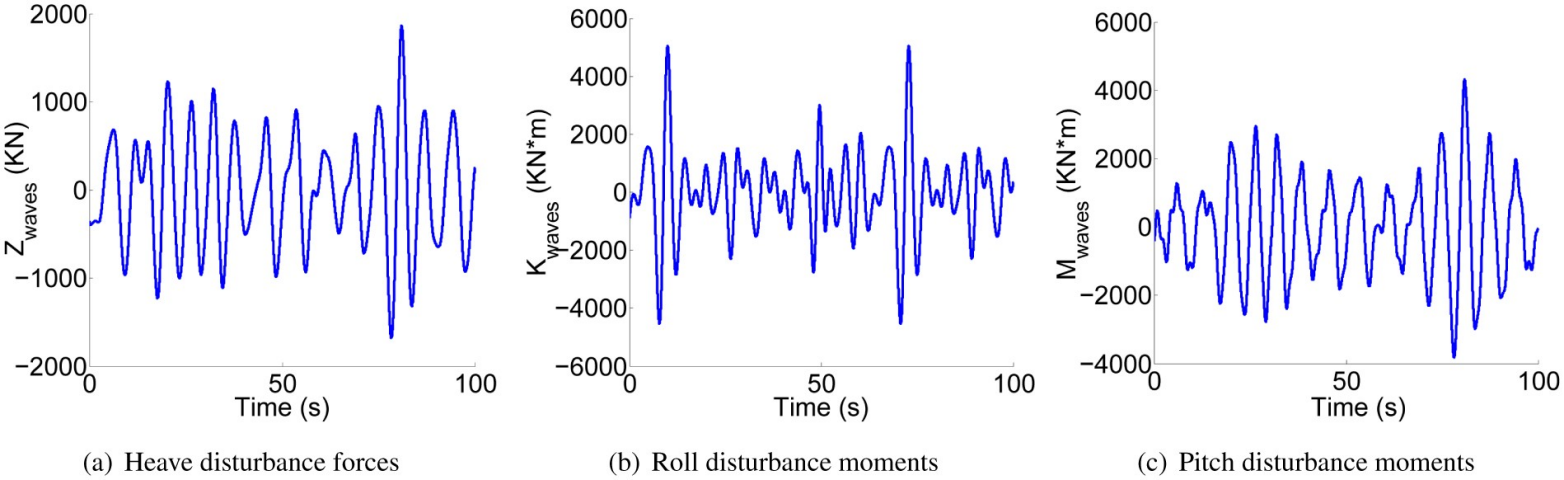
Disturbing forces and moments.

In this paper, the irregular waves are simulated by computer software [27]. Fig 4 shows the heave disturbance force *Z*_*waves*_, roll disturbance moment *K*_*waves*_ and pitch disturbance moment *M*_*waves*_. The irregular disturbance waves are made up of 91 regular wave components. They are described as following:

$$\begin{array}{c} \begin{array}{cc} & Z_{waves}\left(t\right)=\sum_{i=1}^{91}Z_{i}\cos\left(\omega_{i}t+\varphi_{i}\right)\\ & K_{waves}\left(t\right)=\sum_{i=1}^{91}K_{i}\cos\left(\omega_{i}t+\varphi_{i}\right)\\ & M_{waves}\left(t\right)=\sum_{i=1}^{91}M_{i}\cos\left(\omega_{i}t+\varphi_{i}\right) \end{array} \end{array}$$ (4)

Where *Z*_*i*_, *K*_*i*_ and *M*_*i*_ are the heave force, roll moment and pitch moment produced by the *i* th wavelet disturbance to the WPC hull, respectively. The constant wave amplitude of each regular wave component *ζ*_*i*_ and the resultant wave *ζ*(*t*) are calculated by:

$$\begin{array}{c} \zeta_{i}=\sqrt{2S(\omega_{i})\Delta \omega } \end{array}$$ (5)

and

$$\begin{array}{c} \zeta \left(t\right)=\sum_{i=1}^{n}\zeta_{i}\cos\left(\omega_{i}t+\varphi_{i}\right) \end{array}$$ (6)

Where the subscript *i* represents the *i*th regular wave, *ω* is the wave frequency, Δ*ω* is the wave frequency interval, *φ* is the random phase angle, and it is chosen to be a stochastic variable with uniform distribution on the interval [0, 2*π*]. In our study, the Pierson-Moskowits (PM) spectrum is adopted and is given by the following expression:

$$\begin{array}{c} S\left(\omega \right)=\frac{8.1\times 10^{-3}g^{2}}{\omega^{5}}\exp\left(\frac{-3.11}{\omega^{4}h_{1/3}^{2}}\right) \end{array}$$ (7)

Where *g* is still the acceleration of gravity, *h*_1/3_ is the significant wave height.

**Fig 5:**
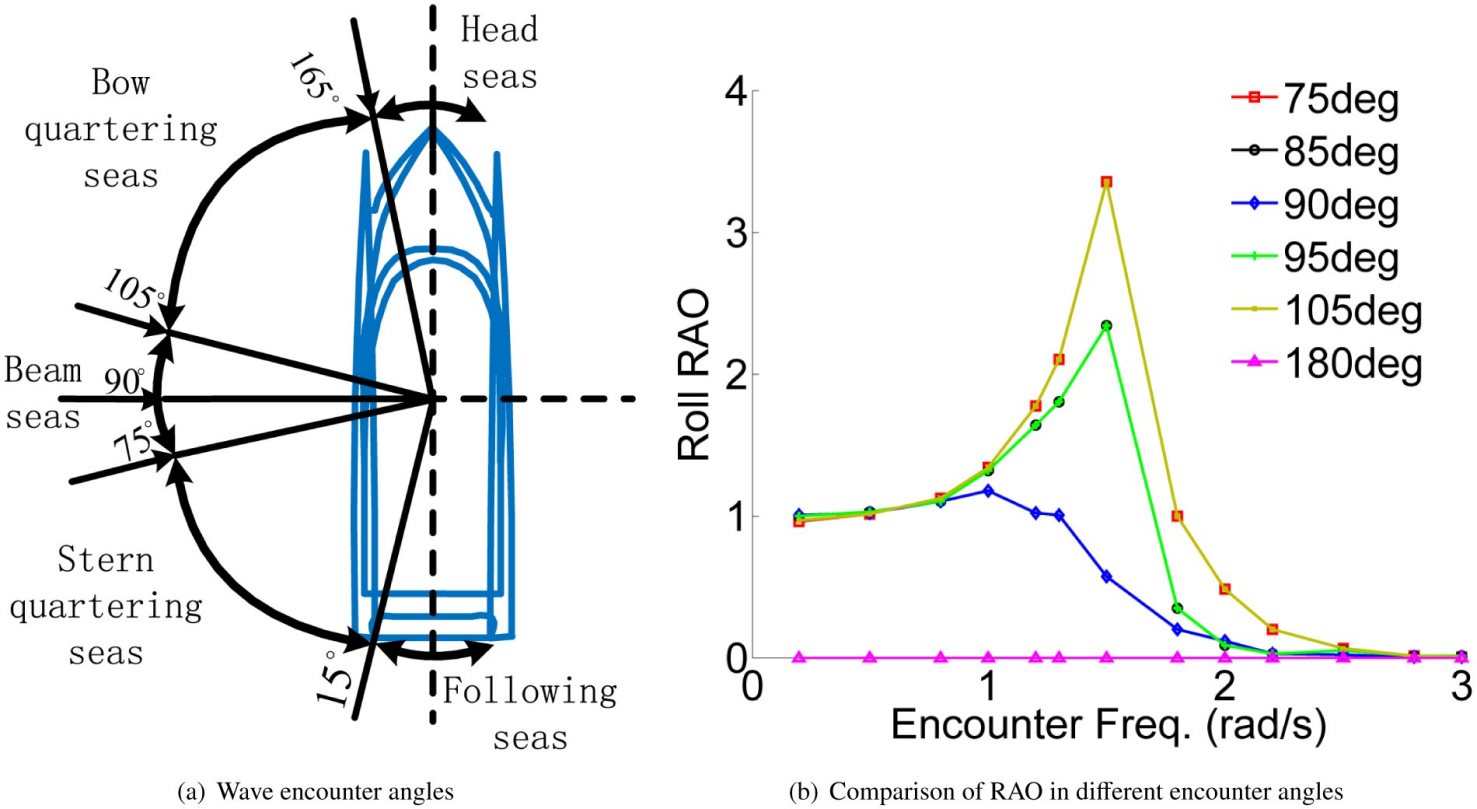
Encounter angles and roll RAO. (a) Wave encounter angles. (b) Comparison of RAO in different encounter angles.

The angle between the wave-propagation direction and the ship course is defined as the wave encounter angle. Fig 5 shows several major waves to the hull and the roll response amplitude operator (RAO) in different encounter angles. The range of 85deg to 95deg is chosen as the main research in this paper. When the encounter angles are 75deg and 105deg, the roll motion is very violent and it is difficult to control for the WPC vessel, because these two angles are in the quartering seas. And when the encounter angle is 180deg, the roll RAO is always zero as shown in Fig 5(b).

#### 2.2.3 Stern flaps forces and moments

**Fig 6:**
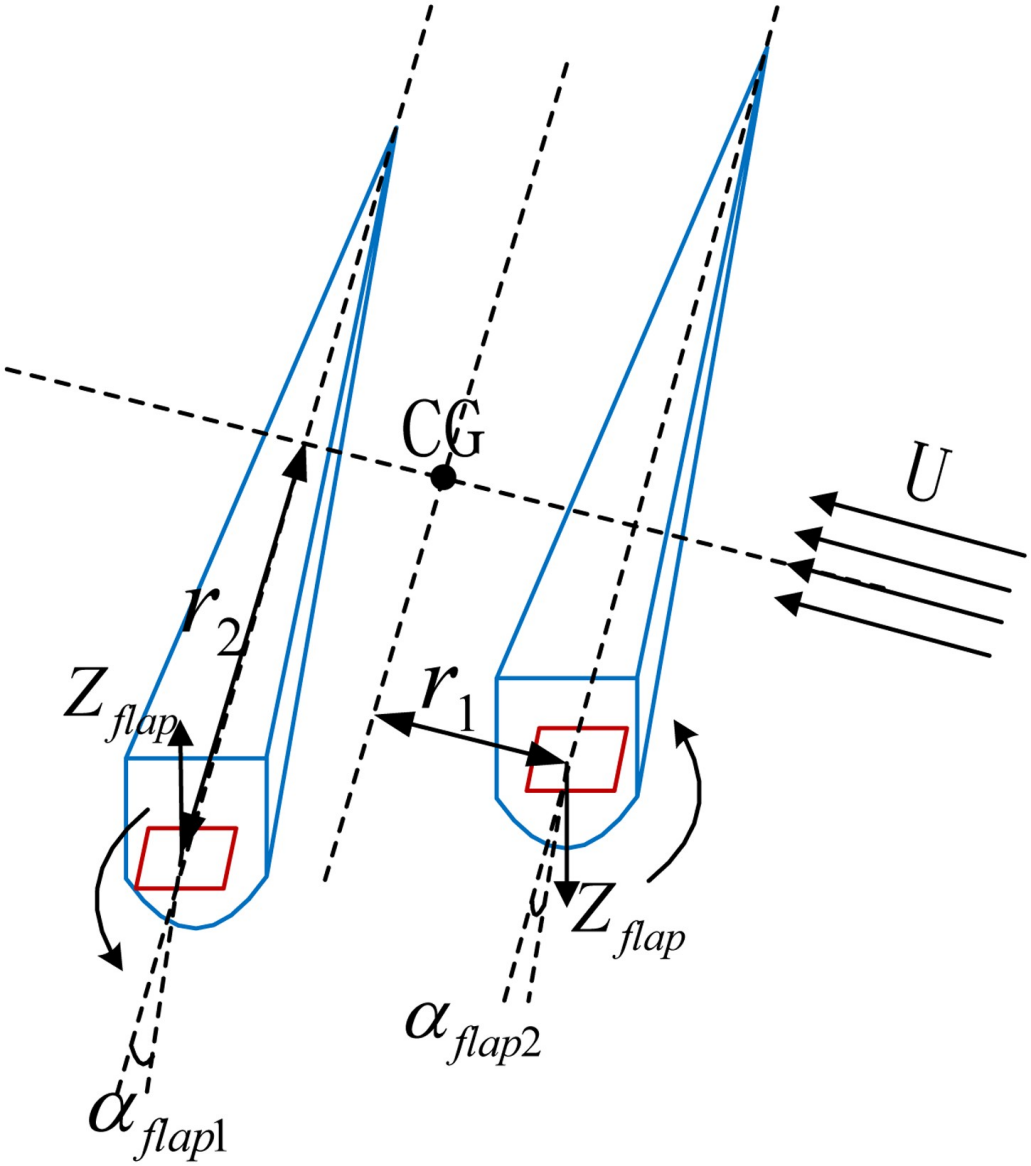
An anti-3 DOF motion system with two flaps.

The stern flap is a typical stabilizer. The pressure on its upper and lower surface varies with the change of the angle of attack. There are two flaps located in the stern and the forces and moments can be obtained by regulating the attack angles in different directions [26]. These forces and moments can simultaneously reduce the heave, roll and pitch motion for the WPC vessel. The force analysis is shown in Fig 6.

The two flaps induced forces and moments [19] can be expressed as:

$$\begin{array}{c} \begin{array}{cc} & Z_{flap}=\frac{1}{2}\rho AU^{2}C_{L}\left(\alpha_{flap}\right)\\ & K_{flap}=K_{flap}\cdot r_{1}\\ & M_{flap}=Z_{flap}\cdot r_{2} \end{array} \end{array}$$ (8)

Where *ρ* is the fluid density, *A* is the area of the flap, *U* is the vessel’s speed, *r*_1_ is the longitudinal distance between the pressure center of the two flaps and the center of gravity (CG) of the WPC vessel, *r*_2_ is the transverse distance between the pressure center of the two flaps and the vessel’s CG, *α*_*flap*_ is the attack angle of the flap and it can rotate in 15deg with respect to the horizontal plane. There are two attack angles for two flaps respectively, one is referred as *α*_*flap*1_ and the other is referred as *α*_*flap*2_. *C*_*L*_(*α*_*flap*_) is the lift coefficient and it can be obtained by dynamically flapping the flaps using computer fluid dynamic (CFD) simulation software.

**Fig 7:**
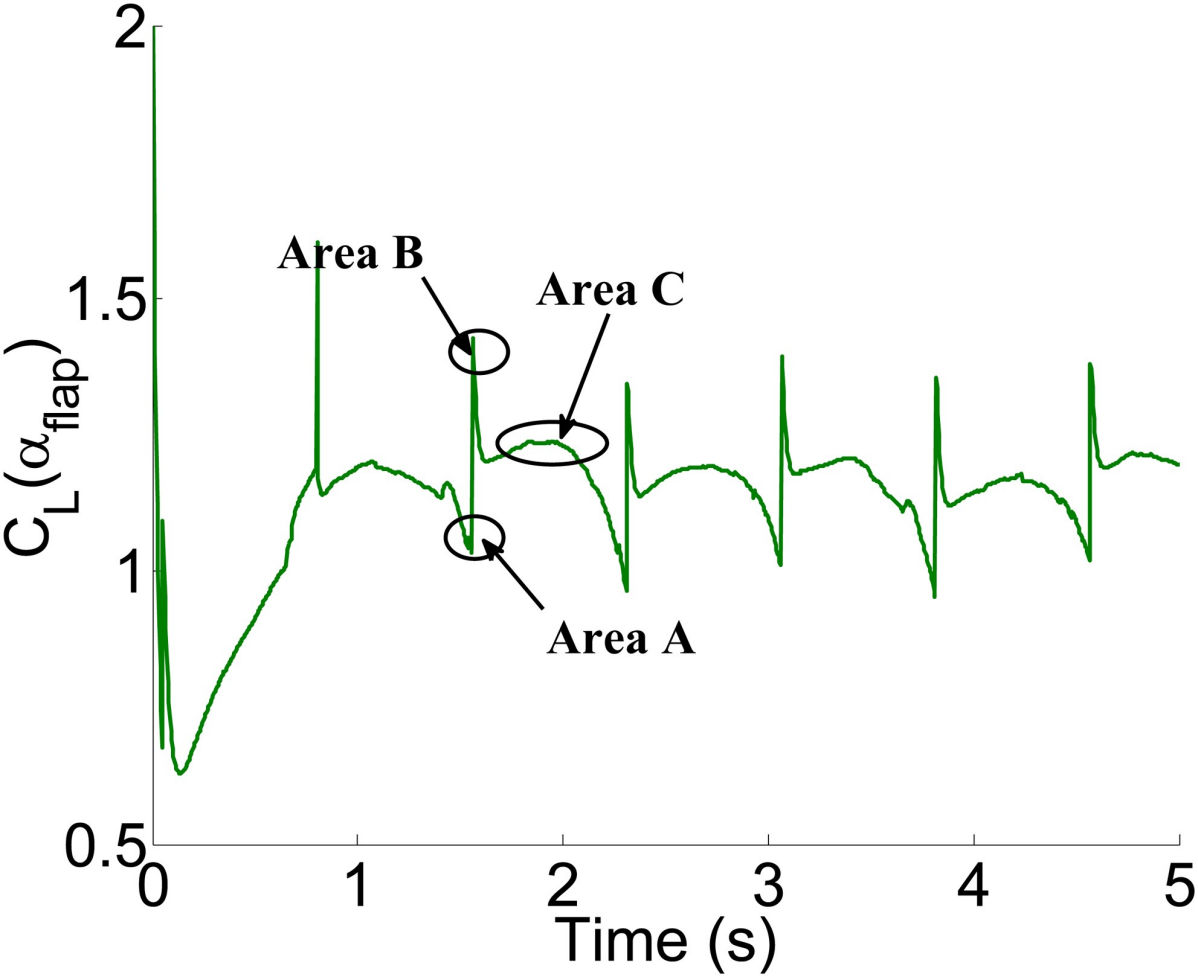
The dynamic lift coefficient curve of two flaps.

Fig 7 shows the dynamic lift coefficient curve under the different flapping directions for two flaps. When the attack angles of the two flaps are zero degree at the same time, the area *A* is the smallest value; At a certain point in the middle, the area *B* is the largest value; When the attack angles are maximum value at the same time, the area *C* is the coefficient value of the final selection.

**Fig 8:**
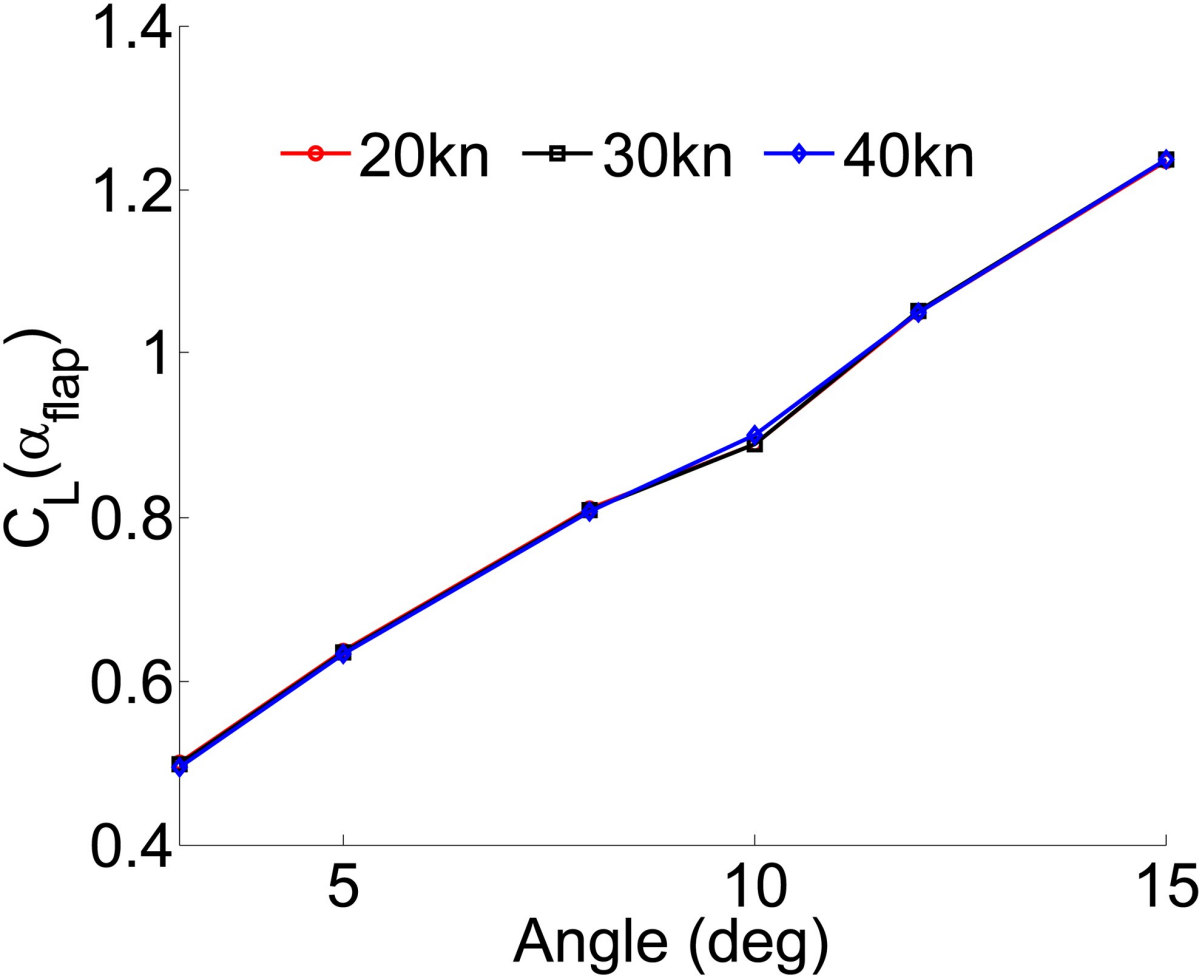
Lift coefficient fitting curves in different speeds.

Fig 8 shows the lift coefficient fitting curves of the flap stabilizer for the WPC vessel. It can be seen that the lift coefficients at different speeds are approximate equality. Thus, the stabilizer lift coefficient is irrelevant to the speeds of the vessel. The fitting equation is described as follow:

$$\begin{array}{c} C_{L}\left(\alpha_{flap}\right)=p_{1}\alpha_{flap}+p_{2} \end{array}$$ (9)

Where *p*_1_ = 0.06086, *p*_2_ = 0.3156.

### 2.3 Ride control system design of WPC

**Fig 9:**
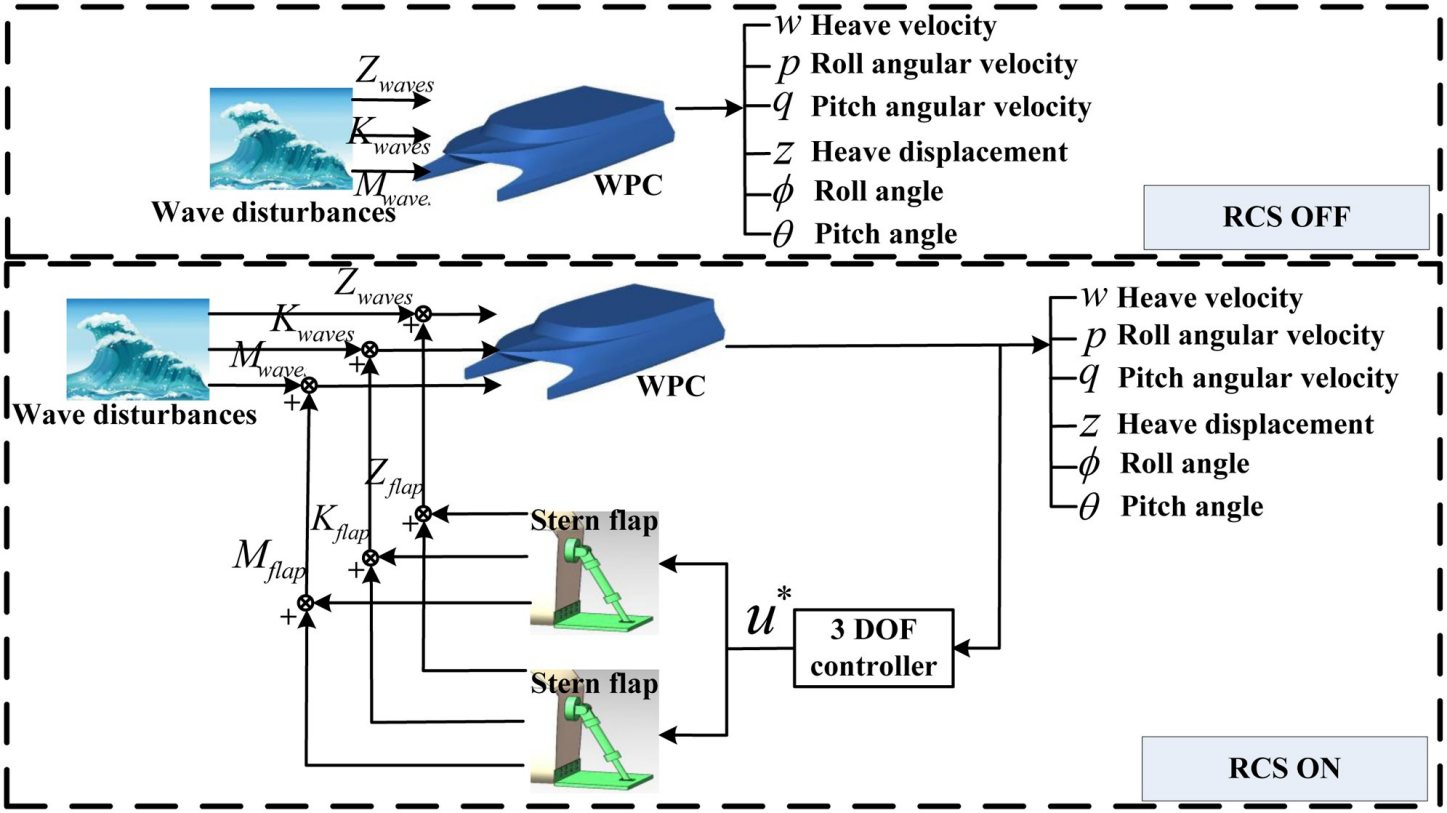
Ride control system ON/OFF for the WPC vessel.

Heave, roll and pitch motion have a great effect on slamming, short-term equipment failure, and the life safety of passengers and crew members for a high-speed vessel. Thus, it is necessary to build a ride control system for a ship with actuators. Fig 9 shows the anti-heave, roll and pitch motion system for the WPC vessel.

There are two main parts in the above block diagram: free vessel and two stern flaps active ride control. There is no RCS in the free vessel part and the heave, roll and pitch motion only induced by wave disturbances. Another part has the RCS with two stern flaps for the WPC vessel, including the free vessel model, actuators model and controller model. And it can be seen that the variable u* is not only the output signal of the proposed controller, but also the input signal of the actuators. The controller gets information from the behaviors of the WPC, and computes two control input data, then the command signals of two flaps are obtained.

However, it is not good for the 3 DOF controller design to establish such a complex nonlinear model (3). Hence, to simplify the RCS design, the state-space model is expressed as follows:

$$\begin{array}{c} \begin{array}{cc} {}[\begin{array}{c} \dot{w}\\ \dot{q}\\ \dot{p}\\ w\\ q\\ p \end{array}]= & [\begin{array}{cc} -A_{a}^{-1}B_{b} & -A_{a}^{-1}C_{c}\\ I_{3\times 3} & 0_{3\times 3} \end{array}][\begin{array}{c} w\\ q\\ p\\ z\\ \theta \\ \phi \end{array}]+[\begin{array}{c} A_{a}^{-1}\\ 0_{3\times 3} \end{array}][\begin{array}{c} Z_{waves}\\ M_{waves}\\ K_{waves} \end{array}]d\\ & +[\begin{array}{c} A_{a}^{-1}\\ 0_{3\times 3} \end{array}][\begin{array}{c} Z_{flap}\\ M_{flap}\\ K_{flap} \end{array}]u \end{array} \end{array}$$ (10)

Where $\left[A_{a}\right]=[\begin{array}{ccc} m-Z_{\dot{w}} & Z_{\dot{q}} & 0\\ M_{\dot{w}} & I_{yy}-M_{\dot{q}} & 0\\ 0 & 0 & I_{xx}-K_{\dot{p}} \end{array}]$, $\left[B_{b}\right]=[\begin{array}{ccc} Z_{w} & Z_{q} & 0\\ M_{w} & M_{q} & 0\\ 0 & 0 & K_{p} \end{array}]$, $\left[C_{c}\right]=[\begin{array}{ccc} Z_{z} & Z_{\theta } & 0\\ M_{z} & M_{\theta } & 0\\ 0 & 0 & K_{\phi } \end{array}]$, *d* is the disturbance signal inputs, and *u* is the control signal inputs. Let the state variables be *x* = [*w*, *q*, *p*, *z*, *θ*, *ϕ*]^*T*^, and the output variables be *y* = *x*, $A=[\begin{array}{cc} -A_{a}^{-1}B_{b} & -A_{a}^{-1}C_{c}\\ I_{3\times 3} & 0_{3\times 3} \end{array}]$, $B_{1}=[\begin{array}{c} A_{a}^{-1}\\ 0_{3\times 3} \end{array}][\begin{array}{c} Z_{waves}\\ M_{waves}\\ K_{waves} \end{array}]$, $B_{2}=[\begin{array}{c} A_{a}^{-1}\\ 0_{3\times 3} \end{array}][\begin{array}{c} Z_{flap}\\ M_{flap}\\ K_{flap} \end{array}]$.

Then, the state-space equation is given as follows:

$$\begin{array}{c} \begin{array}{cc} & \dot{x}=Ax+B_{2}u+B_{1}d\\ & y=Cx \end{array} \end{array}$$ (11)

Where *A* is the system matrix, *B*_2_ is the control matrix, *B*_1_ is the wave disturbance matrix, and *C* = *I*_6×6_ is the output matrix.

## 3 Controller design

### 3.1 Linear quadratic regulator design

Linear quadratic regulator problem [28–30] is a potent technical means for multiple systems’ controller design (such as linear systems, single input single output (SISO) systems and multiple input multiple output (MIMO) systems etc.). LQR seeks the optimal controller parameters that minimizes the given performance index. The integral of quadratic function of the state vectors and the system input vectors are used as performance index. Then the optimal state feedback control law can be obtained. Hence, the LQR controller is used for our RCS of WPC based on state space expression (11).

And then, we define the following performance index:

$$\begin{array}{c} J=\frac{1}{2}\int_{0}^{\infty }\left(x^{T}Qx+u^{T}Ru\right)dt \end{array}$$ (12)

Where *Q* and *R* are weight matrices, *Q* is a nonnegative definite matrix and is related to the system state variables *x*, and *R* is a positive definite matrix and is related to the control inputs *u*, respectively.

The optimal control inputs u* can be written as:

$$\begin{array}{c} u^{*}=-Kx^{*} \end{array}$$ (13)

Where x* is the optimal state variables, *K* is the feedback gain matrix and it is calculated as:

$$\begin{array}{c} K=R^{-1}B_{2}^{T}P \end{array}$$ (14)

Where *P* is the solution of the Algebraic Riccati Equation (ARE) and the equation is described as following:

$$\begin{array}{c} PA+A^{T}P-PB_{2}R^{-1}B_{2}^{T}P+Q=0 \end{array}$$ (15)

According to Eq (15), we need to find the optimal weighting matrices *Q* and *R* to solve the matrix ARE, and then the optimal control u* is obtained to minimize performance object *J*. When designers randomly fill the matrices *Q* and *R* and they do not follow any general rules, this is called the trial and error method [31]. For the RCS of the multiple degrees of freedom in our study, it is very difficult and time-consuming to use the trial and error method to find the optimal control solution. Hence, to address the selection of *Q* and *R* problem of LQR, an optimization algorithm is incorporated in the LQR controller design framework.

### 3.2 Genetic algorithm

Genetic algorithm is a simulated evolutionary algorithm based on the self-adapting global optimization search, which is formed by the operation of the natural selection mechanism [32–34]. Therefore, main processes for applying GA to get the optimal *Q* and *R* parameters are as follows [35–37], and the SIMULINK software for simulation of the GA was used and was written using a MATLAB programming language [2].

#### 3.2.1 Encoding

The number of elements of *Q* and *R* matrices depends on the number of state variables *x* and the input variables *u*, respectively. Both *Q* and *R* should be symmetric matrices and they are selected as the diagonal matrices usually. Therefore, they are coded as Eq (16) and the quadratic performance index *J* is simply a weighted integral of the square of the states *x* and inputs *u*. Then, Eq (12) can be rewritten as Eq (17).

$$\begin{array}{c} \begin{array}{cc} & Q=diag(q_{11},q_{22},q_{33},q_{44},q_{55},q_{66})\\ & R=diag(r_{11},r_{22}) \end{array} \end{array}$$ (16)

$$\begin{array}{c} \begin{array}{cc} J=\frac{1}{2}\int_{0}^{\infty }( & q_{11}w^{2}+q_{22}q^{2}+q_{33}p^{2}+q_{44}z^{2}+q_{55}\theta^{2}+q_{66}\phi^{2}\\ & +r_{11}\alpha_{flap1}^{2}+r_{22}\alpha_{flap2}^{2})dt \end{array} \end{array}$$ (17)

Where *q*_*ii*_, *i* = 1, 2, …, 6 are the weights of heave velocity, pitch angular velocity, roll angular velocity, heave displacement, pitch angle and roll angle, respectively. Similarly, *r*_*jj*_, *j* = 1, 2 are the weights of two control inputs-two different attack angles of the stern flaps.

#### 3.2.2 Generating initial populations

Firstly, the initial population starts from random population. Hence, we set the initial population size to 100, and elite individual number are 10, and then the random initial populations are obtained by simulation assistance tool.

#### 3.2.3 Selecting fitness function

It is important to select the fitness function for the goal of the WPC RCS control. Therefore, according to heave, roll and pitch motion for the WPC, the fitness function of GA optimization can be described as:

$$\begin{array}{c} \begin{array}{cc} J\left(\kappa \right)=\min_{\kappa \in S} & [d_{1}\cdot \left(\frac{1}{N}\sum_{i=1}^{N}w^{2}\right)+d_{2}\cdot \left(\frac{1}{N}\sum_{i=1}^{N}q^{2}\right)+d_{3}\cdot \left(\frac{1}{N}\sum_{i=1}^{N}p^{2}\right)\\ & +d_{4}\cdot \left(\frac{1}{N}\sum_{i=1}^{N}z^{2}\right)+d_{5}\cdot \left(\frac{1}{N}\sum_{i=1}^{N}\theta^{2}\right)+d_{6}\cdot \left(\frac{1}{N}\sum_{i=1}^{N}\phi^{2}\right)] \end{array} \end{array}$$ (18)

Where *κ* is the parameter vector, *S* is the span of the parameter vector, *N* is the maximum number of generations, *d*_*i*_, *i* = 1, 2, …, 6 is used to balance the control effect of each state variable *x*, However, the fitness function expression (18) is not unique because of the different values of *d*_*i*_. Table 2 shows the different control effects with different *d*_*i*_ values.

**Table 2:** Different control effects in different fitness functions.

*d*_*i*_ value	Heave reduction	Pitch reduction	Roll reduction
*d*_2_ = *d*_5_ = 1	16.0%	88.1%	4.6%
*d*_2_ = *d*_5_ = 10, *d*_4_ = *d*_6_ = 2	18.7%	74.1%	10.4%
*d*_2_ = *d*_5_ = 10, *d*_4_ = *d*_6_ = 5	44.6%	33.2%	41.0%

#### 3.2.4 Generating the next new generations

Selection, crossover and mutation can be chosen to generate new populations and to search optimal value again. In this study, the proportion of cross offspring is 0.4 and the evolutionary algebra is 20.

#### 3.2.5 Iterating termination condition

We set the stop algebra to 20 and the running time is set to 80 seconds in SIMULINK software, and check the iteration termination condition for the fitness function found in the third step. If true, exit the GA. Otherwise, go back to the fourth step.

**Fig 10:**
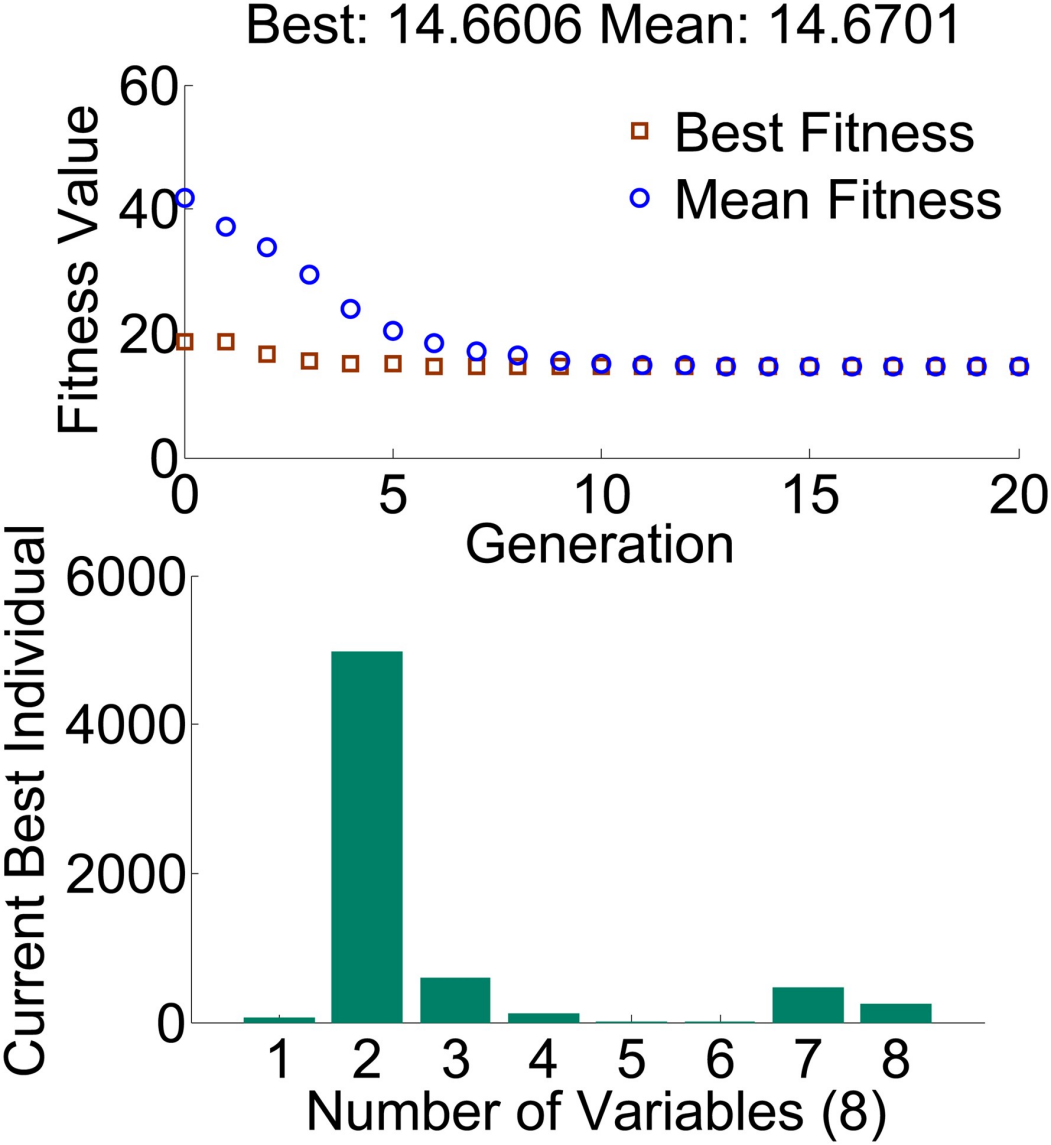
The best GA results.

GA is implemented using simulation software in man-machine interactive way. All of the above steps are also described in Fig 4 of reference [2]. And Fig 10 shows the best GA results, where the value of fitness curve is the result of fitness function and the current best individual is the values of the weighting matrices *Q* and *R*.

**Proof**. The following is the stability analysis of the LQR controller based on GA [2]. Select the following Lyapunov function:

$$\begin{array}{c} V\left(x\right)=x^{T}Px \end{array}$$ (19)

Where for the ∀*x* ≠ 0, there is a *V*(*x*) > 0, and for the ∀‖*x*‖ → ∞, there is a *V*(*x*) → ∞, *P* is still a positive definite symmetric solution of ARE (15). The first-order time derivative of *V*(*x*) is:

$$\begin{array}{c} \dot{V}\left(x\right)={\dot{x}}^{T}Px+x^{T}P\dot{x} \end{array}$$ (20)

Substituting Eqs (11), (13), (14) and (15) into (20) yields:

$$\begin{array}{c} \begin{array}{cc} \dot{V}\left(x\right) & ={\left[\left(A-B_{2}R^{-1}B_{2}^{T}P\right)x\right]}^{T}Px+x^{T}P\left(A-B_{2}R^{-1}B_{2}^{T}P\right)x\\ & =x^{T}\left(A^{T}-P^{T}B_{2}{\left(R^{-1}\right)}^{T}B_{2}^{T}\right)Px+x^{T}PAx-x^{T}PB_{2}R^{-1}B_{2}^{T}Px\\ & =x^{T}A^{T}Px-x^{T}P^{T}B_{2}{\left(R^{-1}\right)}^{T}B_{2}^{T}Px+x^{T}PAx-x^{T}PB_{2}R^{-1}B_{2}^{T}Px\\ & =x^{T}\left(A^{T}P-P^{T}B_{2}{\left(R^{-1}\right)}^{T}B_{2}^{T}P+PA-PB_{2}R^{-1}B_{2}^{T}P\right)x\\ & =x^{T}\left(-Q-P^{T}B_{2}{\left(R^{-1}\right)}^{T}B_{2}^{T}P\right)x\\ & =-x^{T}\left(Q+PB_{2}R^{-1}B_{2}^{T}P\right)x<0 \end{array} \end{array}$$ (21)

According to the Lyapunov stability theorem [38], the proposed controller based on GA stabilizes the system given by Eq (11).

## 4 Results and discussions

### 4.1 3 DOF motion responses

**Fig 11:**
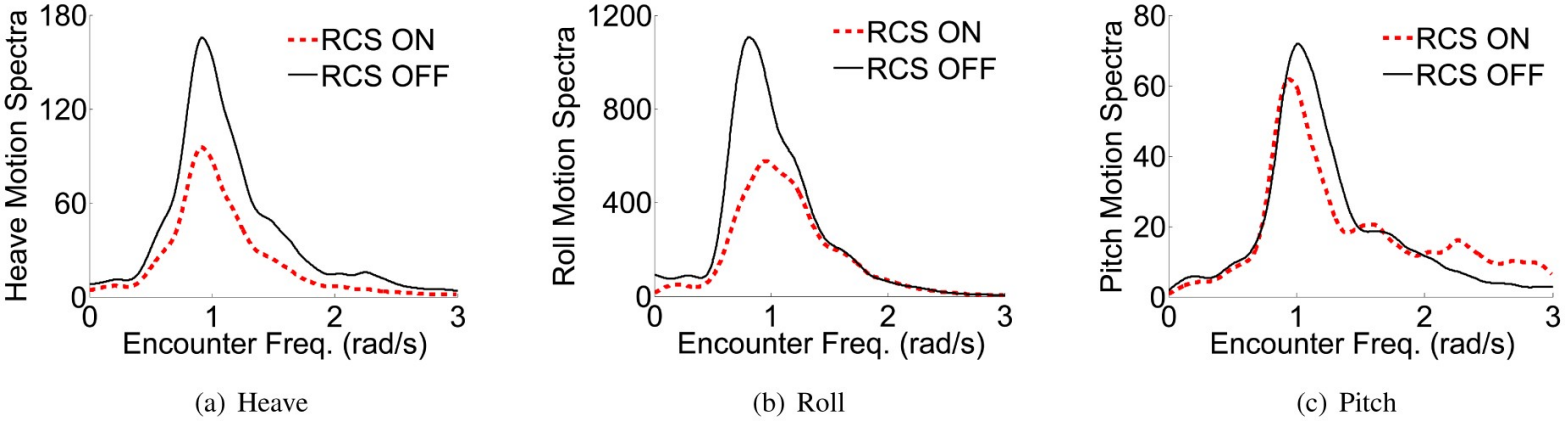
Heave, roll and pitch motion spectra with and without RCS. (a) Heave. (b) Roll. (c) Pitch.

**Fig 12:**
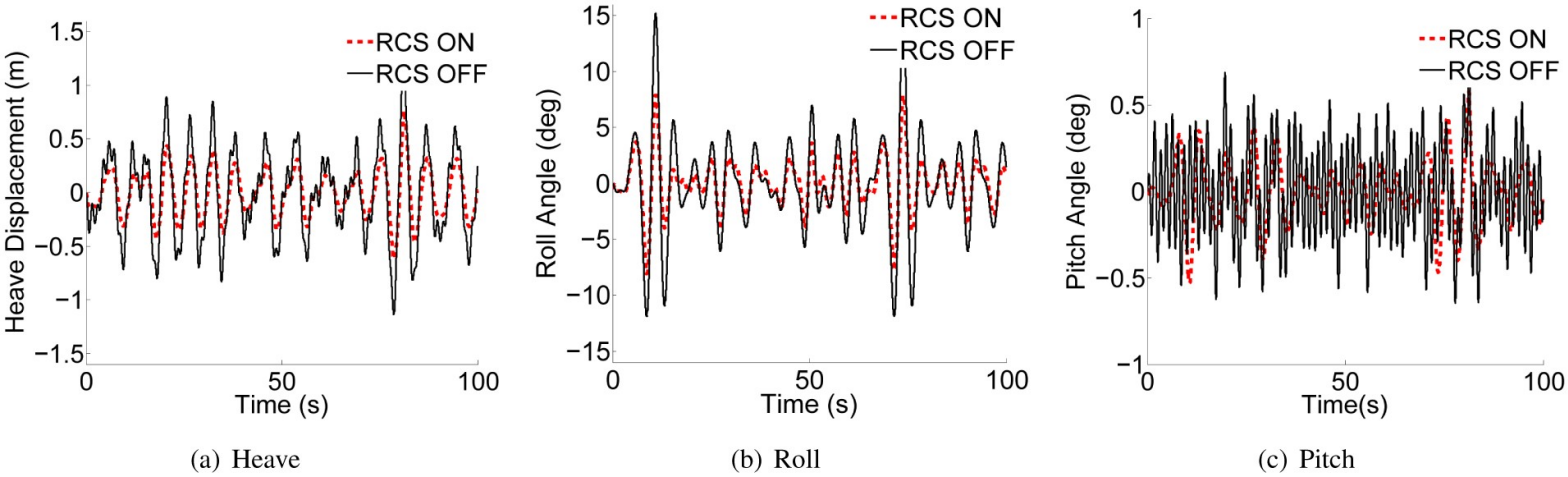
Heave, roll and pitch motion displacements and angles with and without RCS. (a) Heave. (b) Roll. (c) Pitch.

Perez et al. [39, 40] described the ship motion responses in time and frequency domain respectively. Based on this, to illustrate the effectiveness of the RCS with two flaps based on LQR-GA method, the comparisons of RCS ON and RCS OFF for the heave, roll and pitch motion responses in frequency domain and time domain separately are shown in Figs 11 and 12. The results were obtained when the ship is sailing at 40kn, SSN4 and beam seas. The plots of the motion spectra in Fig 11 clearly illustrate that the motion spectrum of heave, roll and pitch reaches the maximum at a certain corresponding frequency point, and it can be seen that the motion power spectra are all obviously reduced after adding the proposed controller. While the WPC vessel is sailing with beam seas, roll reduction is preferred consideration in this case. Hence, it is critical to trade-off the reduction of heave, roll and pitch. From Fig 12, the heave is reduced 44.6% on average, the roll is reduced 41.0% on average, and the pitch is reduced 33.2% on average. Obviously, the heave and roll reductions are better than that of the pitch. To sum up, Figs 11 and 12 can well illustrate the feasibility and effectiveness of the proposed controller. Consequently, the weight coefficients *Q* and *R* can be obtained by GA method, and the solutions *P* and *K* can be obtained from ARE as shown in Table 3.

**Table 3:** The results of LQR based GA method.

Parameters of system	LQR-GA
Weighting matrix (*Q*)	$\left[\begin{array}{cccccc} 63.9984 & 0 & 0 & 0 & 0 & 0\\ 0 & 4984.7 & 0 & 0 & 0 & 0\\ 0 & 0 & 589.2208 & 0 & 0 & 0\\ 0 & 0 & 0 & 119.0406 & 0 & 0\\ 0 & 0 & 0 & 0 & 5 & 0\\ 0 & 0 & 0 & 0 & 0 & 1 \end{array}\right]$
Weighting matrix (*R*)	$\left[\begin{array}{cc} 469.6317 & 0\\ 0 & 243.1295 \end{array}\right]$
Solution of ARE (*P*)	$\left[\begin{array}{cccccc} 1.1 & 2.9 & -3 & 1.3 & -4.4 & 0.2\\ 2.9 & 148 & -151.5 & 1.7 & -197.4 & 9.5\\ -3 & -151.5 & 491.7 & 2 & 839.5 & -59.6\\ 1.3 & 1.7 & 2 & 90.1 & 18.8 & -1\\ -4.4 & -197.4 & 839.5 & 18.8 & 325.65 & -596.3\\ 0.2 & 9.5 & -59.6 & -1 & -596.3 & 120.7 \end{array}\right]$
Optimal gain (*K*)	$\left[\begin{array}{cccccc} -0.2900 & -1.7423 & -0.4483 & -0.3387 & -1.7748 & 0.2250\\ 0.3176 & -3.4115 & -0.8108 & 0.3514 & -3.7975 & 0.4048 \end{array}\right]$

**Fig 13:**
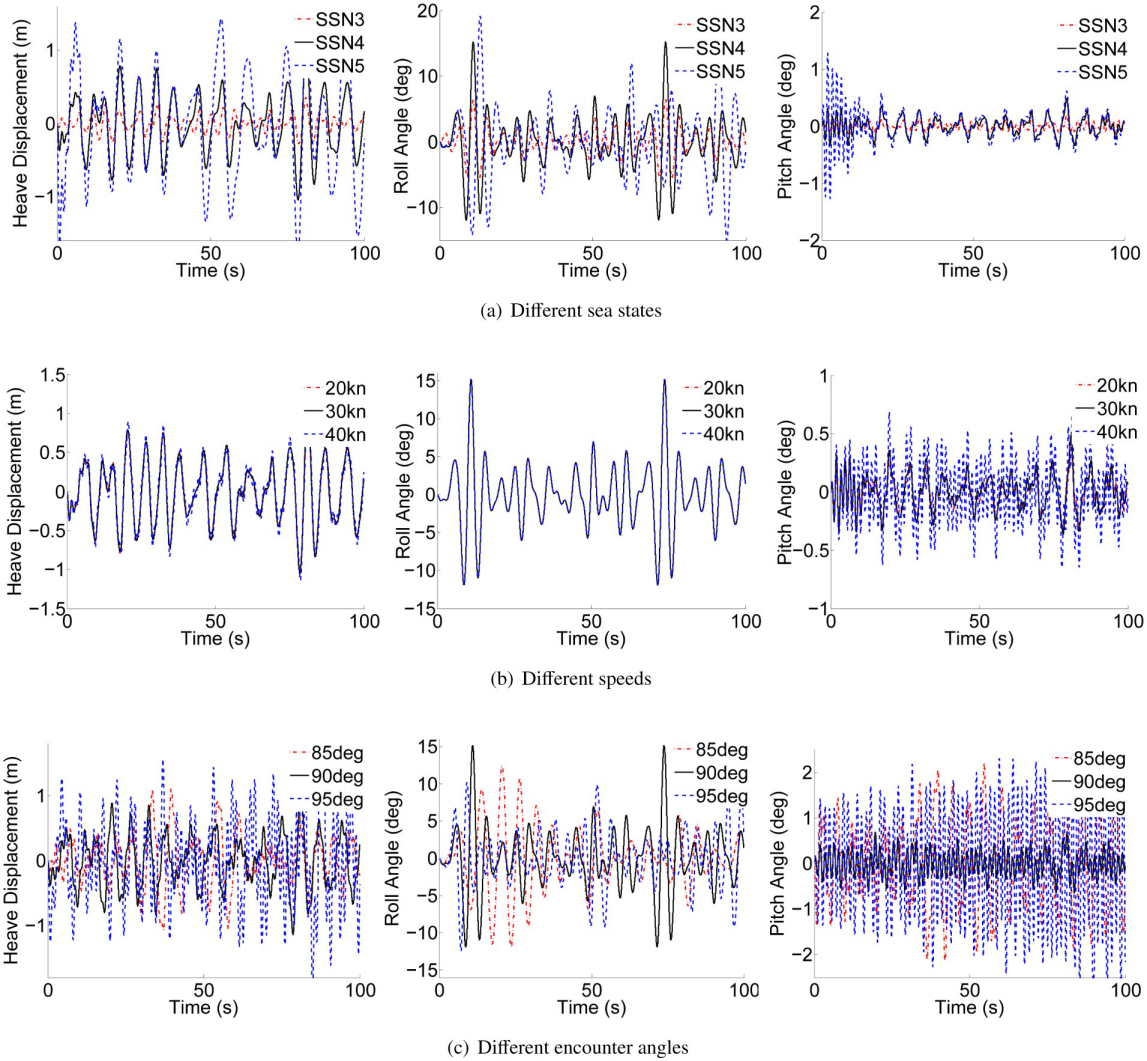
Vibration of the WPC vessel without proposed controller in different situations. (a) Different sea states. (b) Different speeds. (c) Different encounter angles.

Fig 13 shows the heave, roll and pitch amplitudes without proposed controller in different speeds, sea states and encounter angles. It can be seen clearly that the heave and pitch motion are severe when the WPC vessel is sailing at 40kn. However, there is smaller influence on the roll motion when the ship speed changes. Additionally, the 3 DOF motion are becoming more and more violent with the increase of sea states. From Fig 13(c), the heave and pitch motion responses are more violent when the encounter angle is not 90deg. However, the roll motion responses are violent when the encounter angle is in the range of beam seas. Therefore, 3 DOF motion of the WPC vessel are considered in beam seas in this paper. Due to the practical waves are not pure 90deg, so we choose the range of 85deg to 95deg to simulate the ship motion responses.

To investigate the effectiveness of RCS with two flaps, the anti-heave, roll and pitch effects [41] in Table 4 is calculated as follows:

$$\begin{array}{c} Reduction\left(\%\right)=\frac{HRPB-HRPA}{HRPB}\times 100\% \end{array}$$ (22)

Where HRPA and HRPB represent the root mean square (RMS) of the heave displacement, roll angle and pitch angle with and without RCS with two flaps, respectively.

**Table 4:** Anti-3 DOF motion effect with two flaps.

3 DOF	Encounter angles [deg]	Speeds [kn]	SSN	RCS OFF	RCS ON	Reduction [%]
Heave	90	30	3	0.1426	0.0249	82.8
	90	30	4	0.5018	0.4385	12.6
	90	30	5	0.9330	0.8253	11.5
	90	20	4	0.5063	0.4567	9.8
	90	40	4	0.5246	0.2906	44.6
	85	40	4	0.5056	0.2925	42.1
	95	40	4	1.4931	0.8691	41.8
Pitch	90	30	3	0.0809	0.0345	57.4
	90	30	4	0.1856	0.1441	22.4
	90	30	5	0.3529	0.2793	20.9
	90	20	4	0.1649	0.1442	12.6
	90	40	4	0.3407	0.2275	33.2
	85	40	4	1.1875	1.0321	13.1
	95	40	4	1.5779	0.4999	68.3
Roll	90	30	3	0.6765	0.6552	3.1
	90	30	4	3.4885	2.4280	30.4
	90	30	5	7.5589	4.6179	38.9
	90	20	4	3.4839	2.5304	27.4
	90	40	4	3.4782	2.0513	41.0
	85	40	4	5.9475	4.1055	31.0
	95	40	4	0.8691	0.4317	50.3

Table 4 presents the anti-heave, roll and pitch effects of stern flaps in different simulation environments. The heave is reduced 34.5% on average, where the maximum and minimum reduction are 82.8% and 9.8%, respectively. And the other vertical motion-pitch is improved 32.1% on average, where the maximum and minimum reduction are 68.3% and 12.6%, respectively. Finally, the roll has an average reduction of 31.4%, where the maximum and minimum reduction are 50.3% and 3.1%, respectively. From Fig 12 and Table 4, the RCS consisting of two flaps is feasible and the proposed controller based GA is effective for the WPC vessel. Each of the motion response is related to the hydrodynamic parameters in a particular case. Hence, the core of this work is to design a controller which can achieve ideal effect for each motion response by adjusting its control parameters. And we need to do a lot of repeated debugging works in order to get the better control effect.

### 4.2 Motion sickness incidence

Motion sickness incidence (MSI) can be regarded as the performance index of comfort evaluation for ships [42, 43]. In this paper, a MSI model for the 3 DOF motion can be written based on O’Halon and Mc Cauley in 1974 [44]. MSI is a weighted result and every waves movement will accumulate to MSI values, so the superposition of the waves amplitude will increase the amplitude of the ship motion, and MSI will also increase, as expressed in the following equation:

$$\begin{array}{c} MSI=100\cdot [0.5+erf\left(\frac{\pm \log(|a_{zx}\left|/g\right)\mp \mu_{MSI}}{0.4}\right)] \end{array}$$ (23)

Where *erf* is the error function, *a*_*zx*_ is the vertical and roll acceleration in a chosen place averaged over a half motion cycle, and *μ*_*MSI*_ is:

$$\begin{array}{c} \mu_{MSI}=-0.819+2.32{\left(\log_{10}\omega_{e}\right)}^{2} \end{array}$$ (24)

Where *ω*_*e*_ is the dominant frequency of encounter with waves.

**Fig 14:**
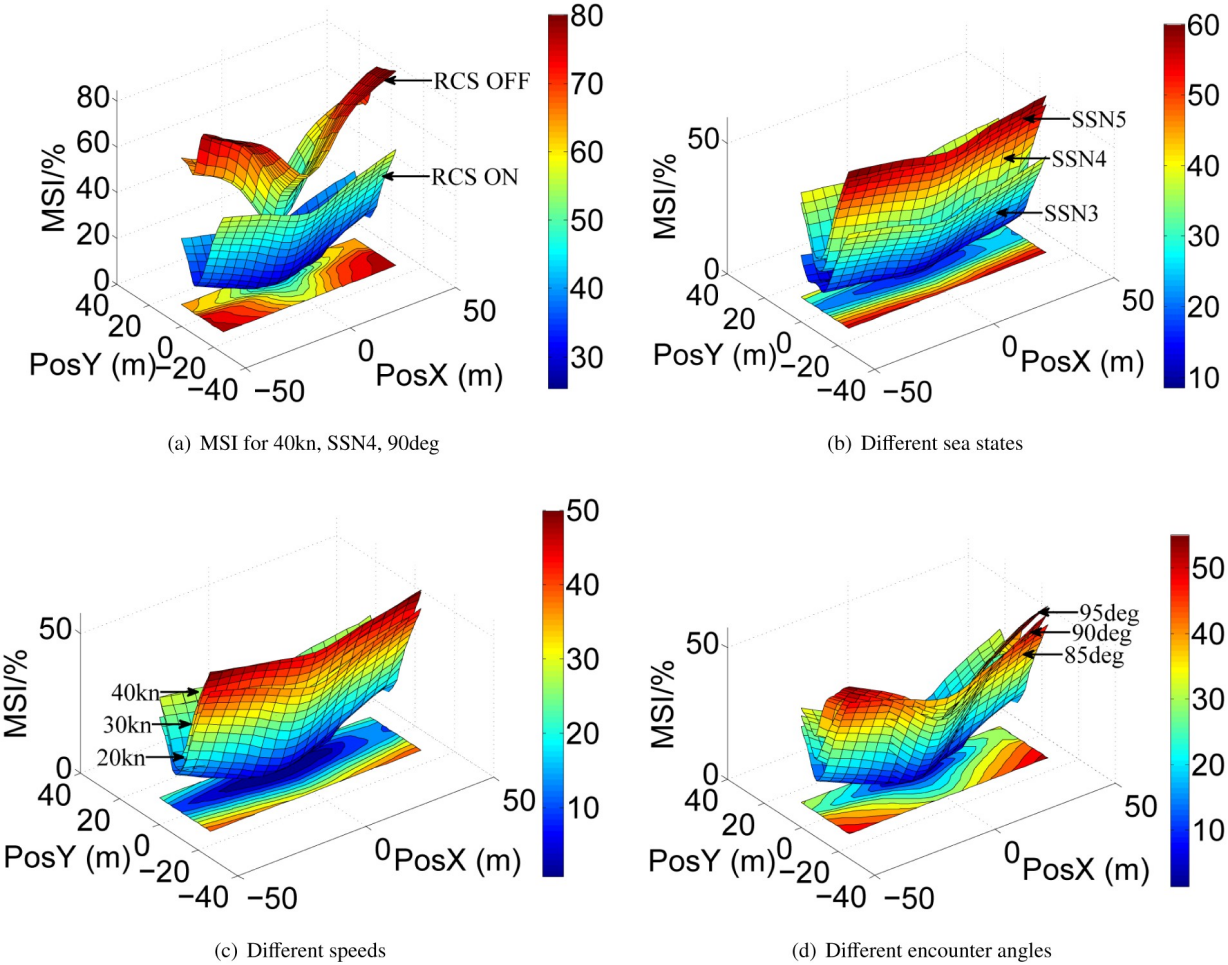
MSI for the WPC vessel in different conditions. (a) MSI for 40kn, SSN4, 90deg. (b) Different sea states. (c) Different speeds. (d) Different encounter angles.

Fig 14 shows the MSI occurring probability when WPC sails at different ocean environments. It can be seen that the MSI results at the stern, bow and sides of the vessel are higher than that at the center. The MSI value with two flaps based on LQR-GA is much smaller than that without the flaps (see Fig 14(a)). Therefore, the validity of the proposed controller is verified again. And with the increase of sea state, speed and encounter angle, the MSI values are larger as shown in Fig 14(b), 14(c) and 14(d).

## 5 Conclusion

1. The 6 DOF dynamics for a ship is characterized by strong nonlinearities and uncertainties because of the complex ocean environment. For the WPC vessel, a simplified 3 DOF (heave, roll and pitch) motion model is built ignoring the smaller hydrodynamic coefficients and the higher order components in beam seas. It is the key to get the hydrodynamic coefficients of the 3 DOF model and find the flapping way to reduce the longitudinal and transverse motion simultaneously.
2. The alternately flapping way of two flaps stabilizers for the 3 DOF stabilization of the WPC vessel is proposed and the selected lift coefficient area of two flaps is efficient to reduce the 3 DOF motion of the vessel.
3. A multiple ride control system (MRCS) for a ship with actuators is built. The LQR-GA control strategy provides an effective approach to reduce the M DOF motion. Depending on robust search mechanisms and global optimum of GA, the optimal control inputs are obtained by choosing proper weighting parameters. Consequently, simulation results illustrate the validity of the RCS for the WPC vessel, and the stability of the proposed controller is proven.
4. In severe waves, it is a difficult problem how to trade-off the heave, roll and pitch motion. All combinations of WPC’s sea states (SSN3, SSN4, and SSN5), speeds (20kn, 30kn, and 40kn) and encounter angles (85deg, 90deg, 95deg) are studied to present the motion state of the WPC vessel in beam seas and verify the correctness of the proposed flapping mode and algorithm. Finally, MSI results demonstrate the feasibility and effectiveness of the proposed controller.
5. Further work with a real WPC vessel test is our next plan and the 6 DOF model will be studied. We expect that our solutions lay a foundation for many researches in this field and come up with more different solutions for conducting maritime tasks.

## Supporting information

S1 Fig Standard notation and sign conventions for a ship motion description. (TIF)

S2 Fig The longitudinal hydrodynamic parameters in different situations. (TIF)

S3 Fig Free roll motion of the WPC vessel. (TIF)

S4 Fig Disturbing forces and moments. (TIF)

S5 Fig Encounter angles and roll RAO. (TIF)

S6 Fig An anti-3 DOF motion system with two flaps. (TIF)

S7 Fig The dynamic lift coefficient curve of two flaps. (TIF)

S8 Fig Lift coefficient fitting curves in different speeds. (TIF)

S9 Fig Ride control system ON/OFF for the WPC vessel. (TIF)

S10 Fig The best GA results. (TIF)

S11 Fig Heave, roll and pitch motion spectra with and without RCS. (TIF)

S12 Fig Heave, roll and pitch motion displacements and angles with and without RCS. (TIF)

S13 Fig Vibration of the WPC vessel without proposed controller in different situations. (TIF)

S14 Fig MSI for the WPC vessel in different conditions. (TIF)

S1 Table Notations used for a ship. (TIF)

S2 Table Different control effects in different fitness functions. (TIF)

S3 Table The results of LQR based GA method. (TIF)

S4 Table Anti-3 DOF motion effect with two flaps. (TIF)
