# Supplementary figures and images for: Design ride control system using two stern flaps based 3 DOF motion modeling for wave piercing catamarans with beam seas
Source: PLoS One. 2019 Mar 25;14(3):e0214400. doi: 10.1371/journal.pone.0214400 (PMC6433233; doi:10.1371/journal.pone.0214400)

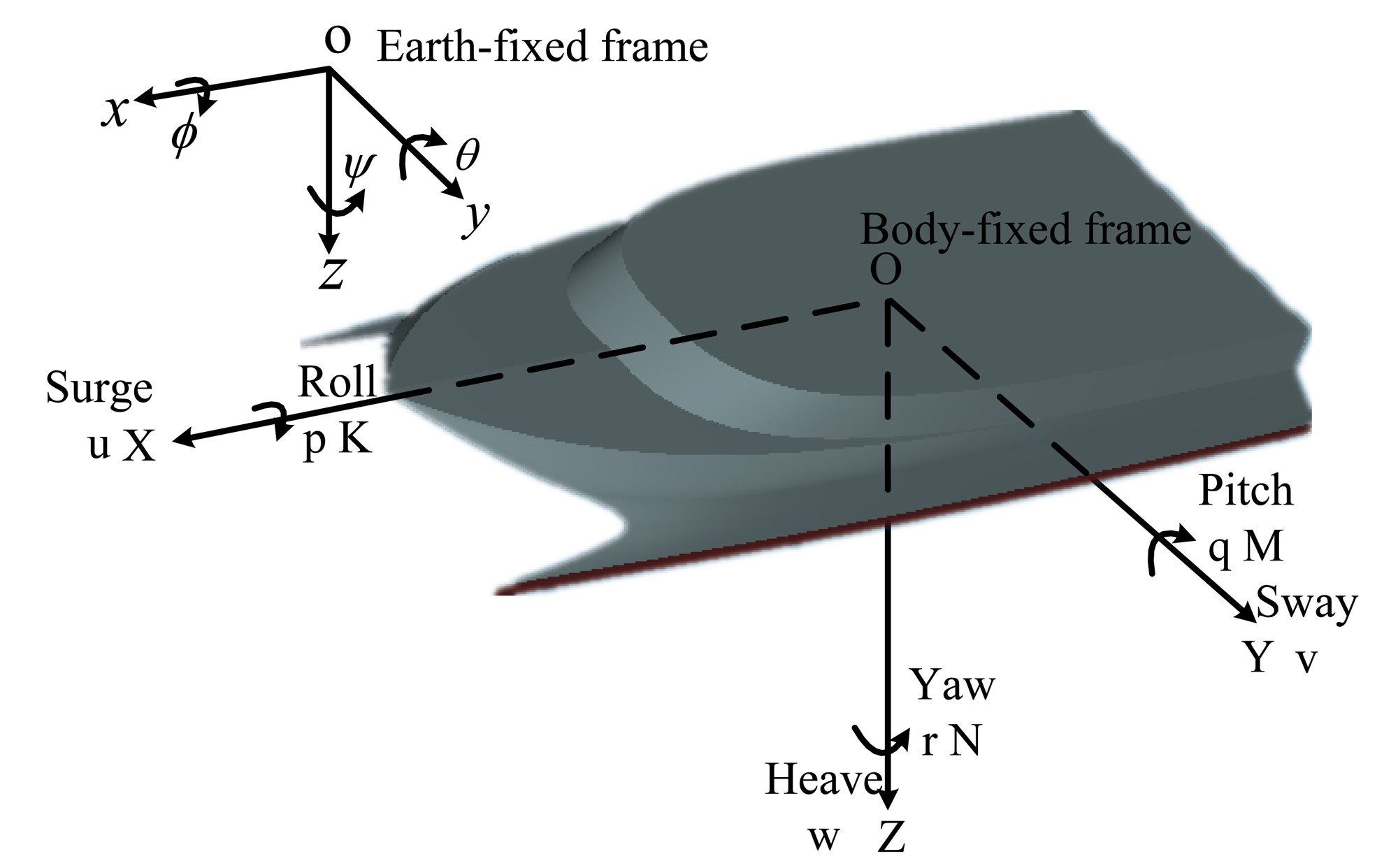

Supplement: S1 Fig — (TIF) [file pone.0214400.s001.tif]

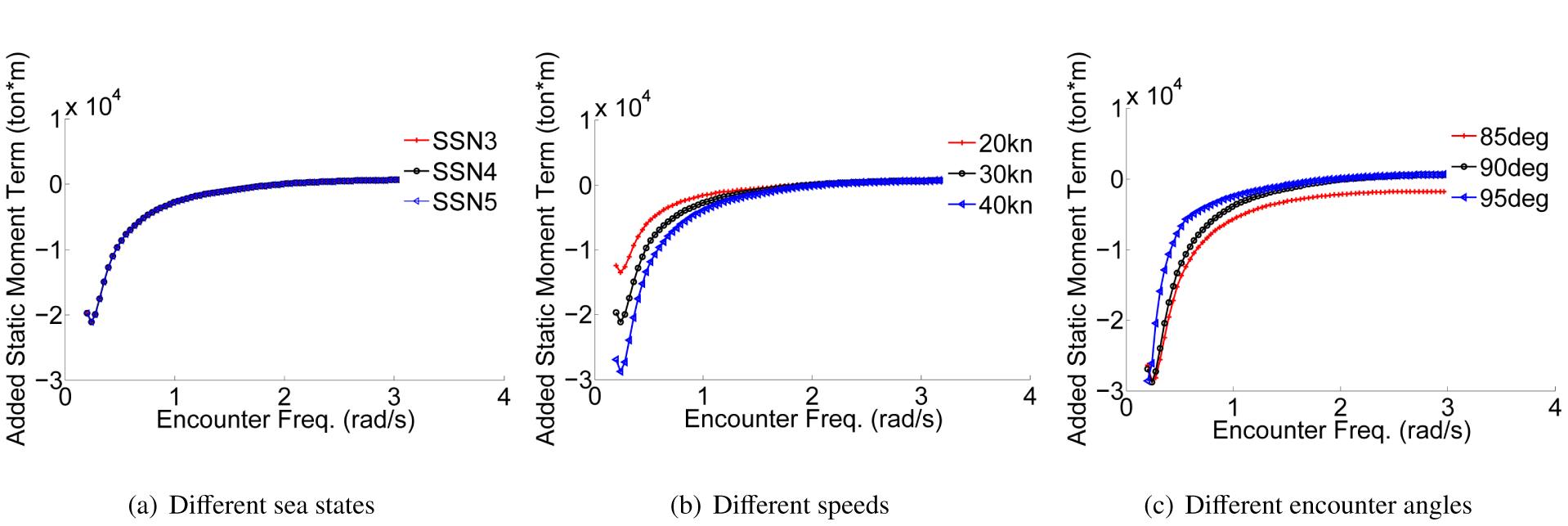

Supplement: S2 Fig — (TIF) [file pone.0214400.s002.tif]

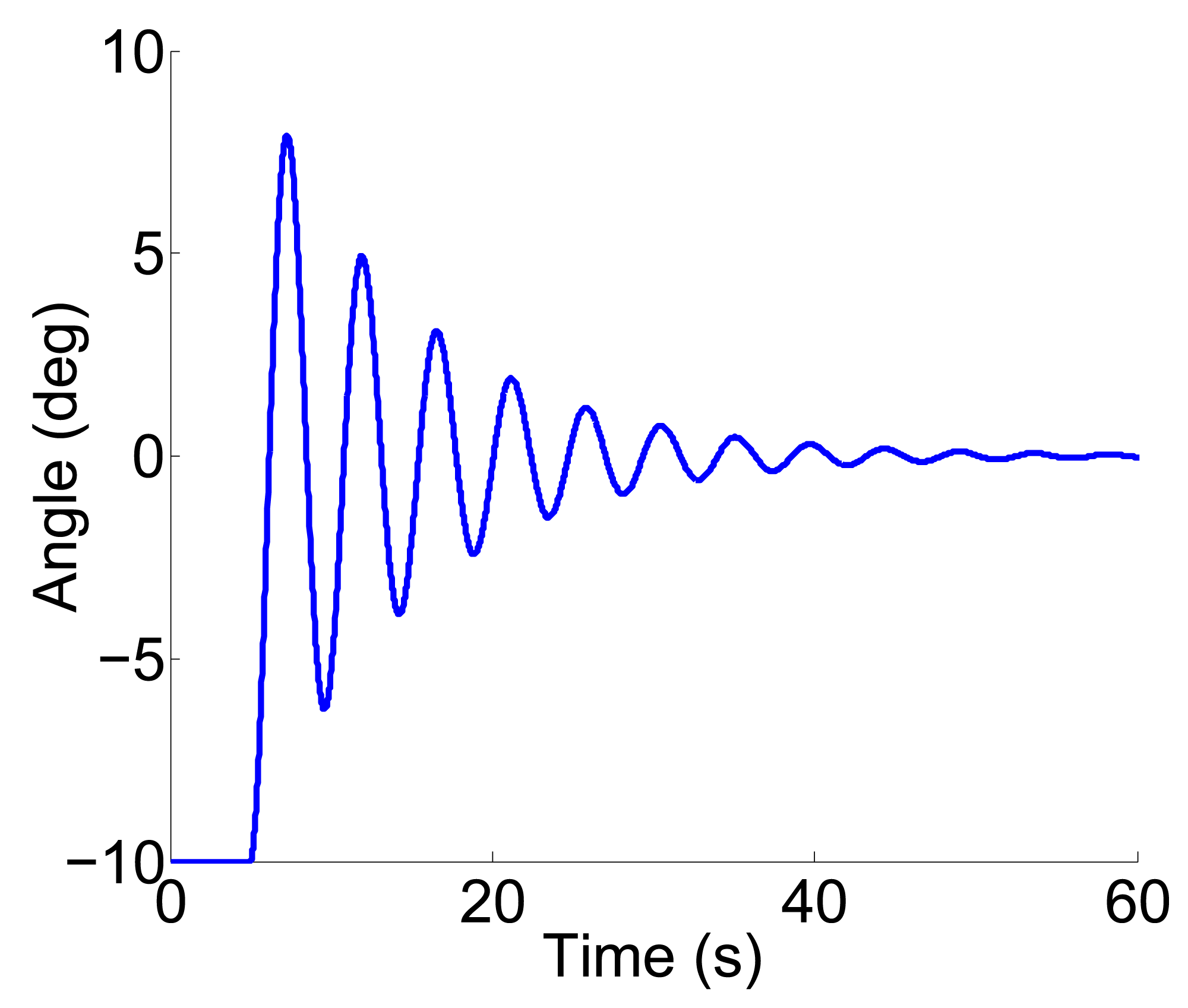

Supplement: S3 Fig — (TIF) [file pone.0214400.s003.tif]

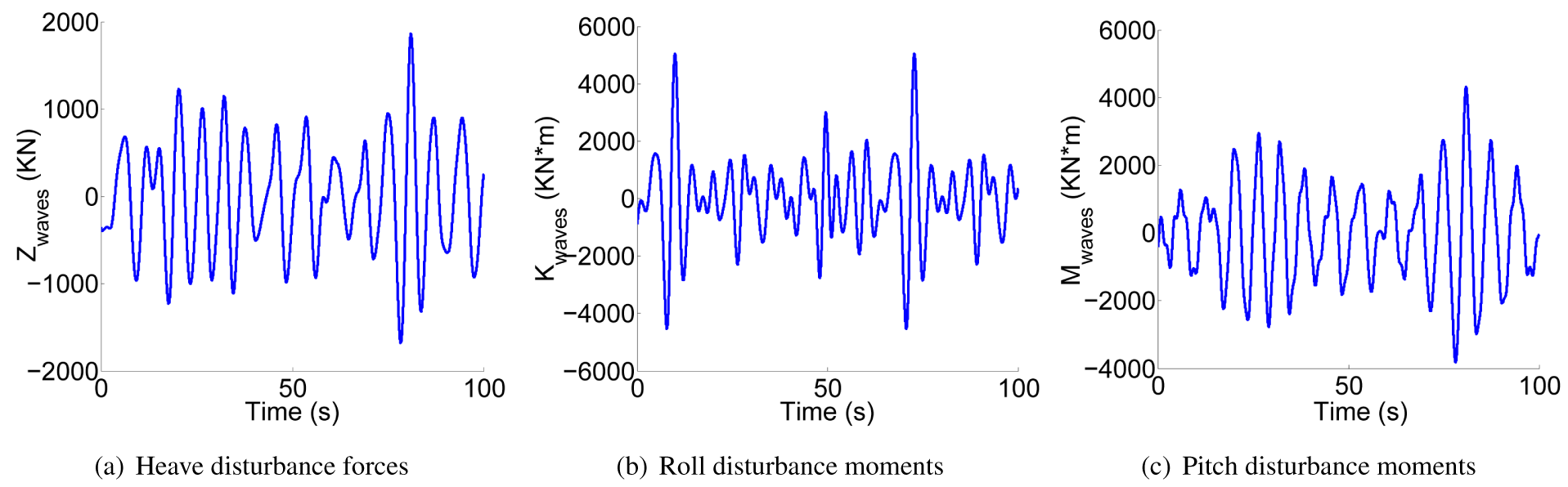

Supplement: S4 Fig — (TIF) [file pone.0214400.s004.tif]

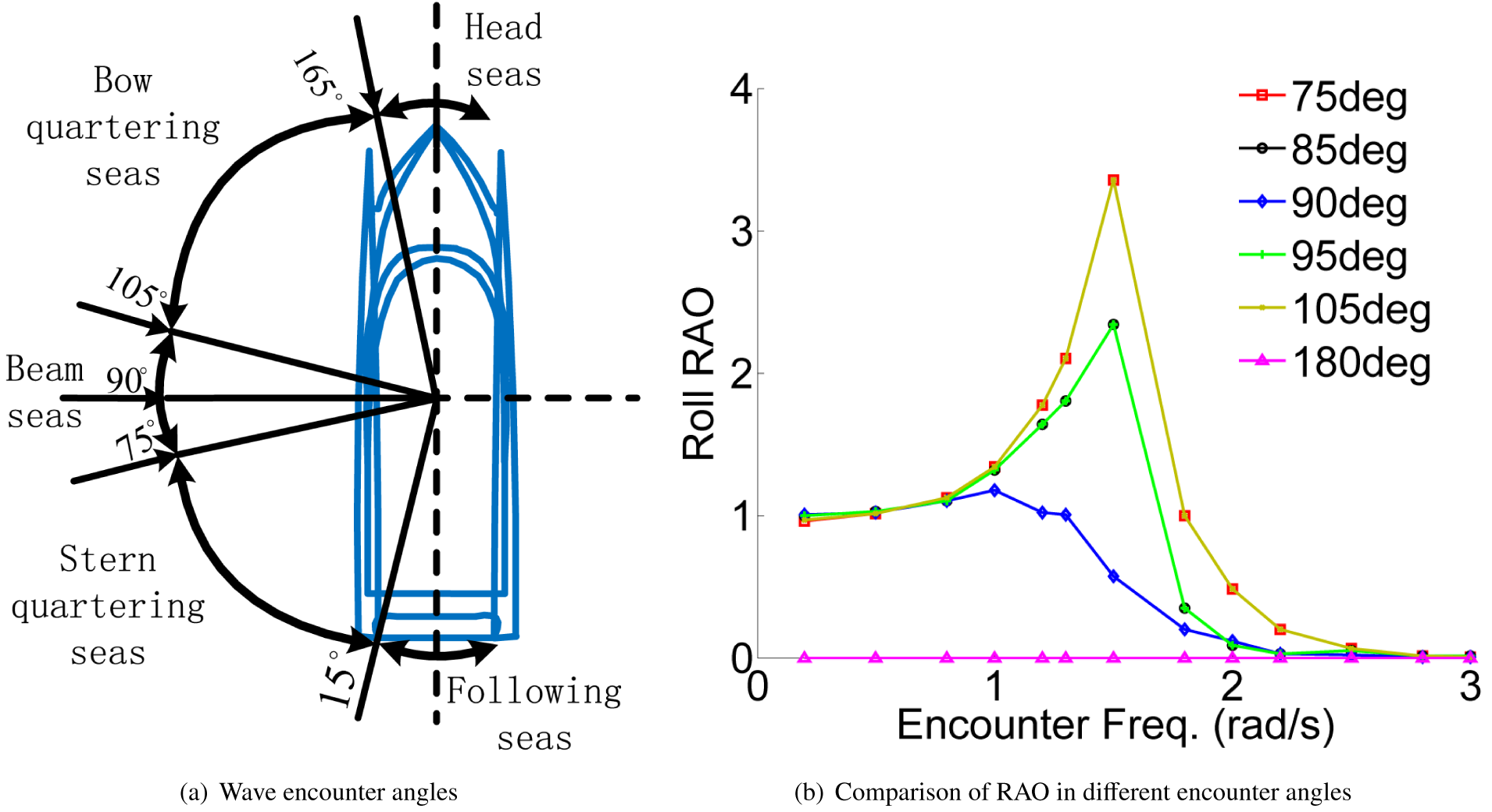

Supplement: S5 Fig — (TIF) [file pone.0214400.s005.tif]

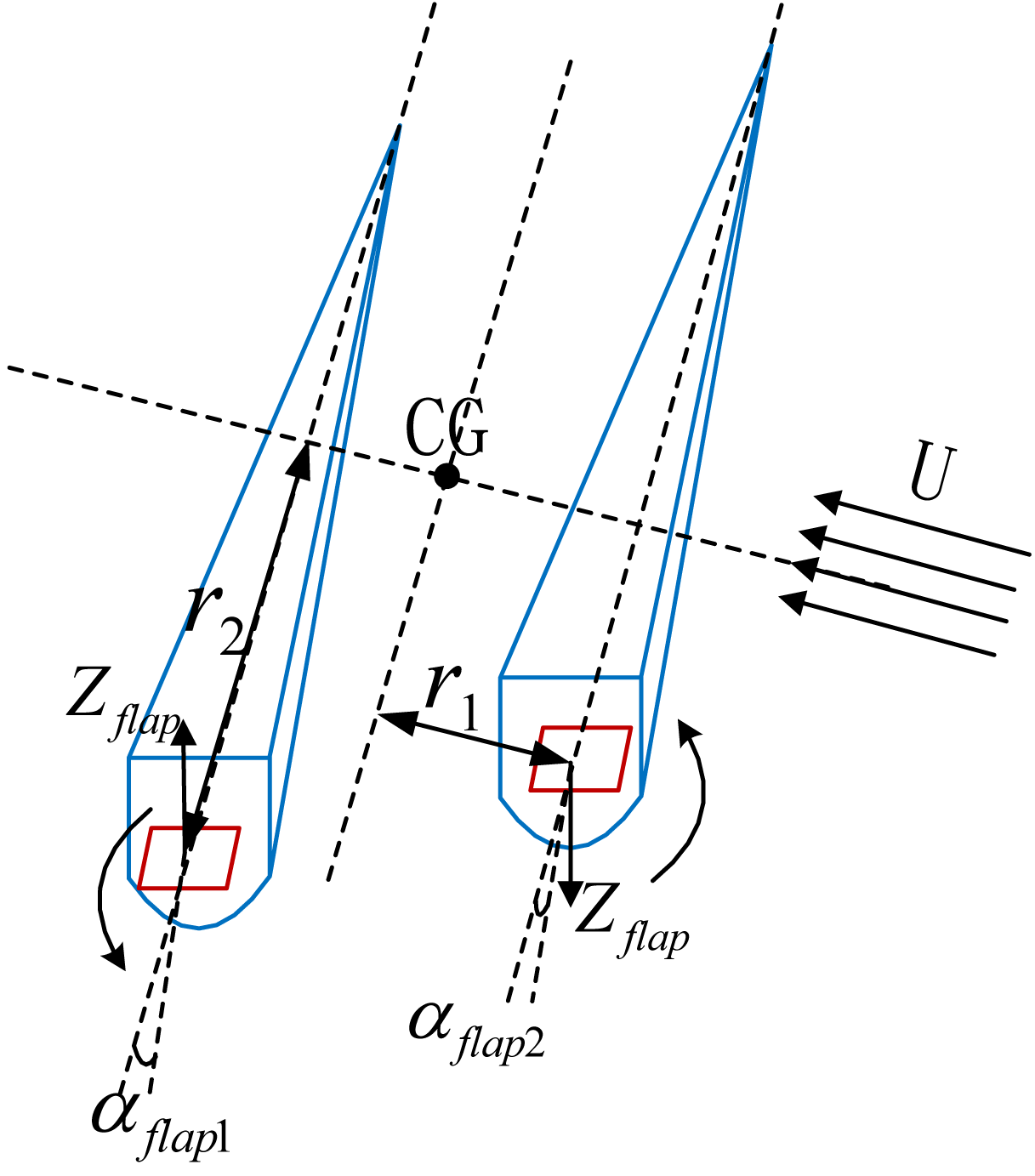

Supplement: S6 Fig — (TIF) [file pone.0214400.s006.tif]

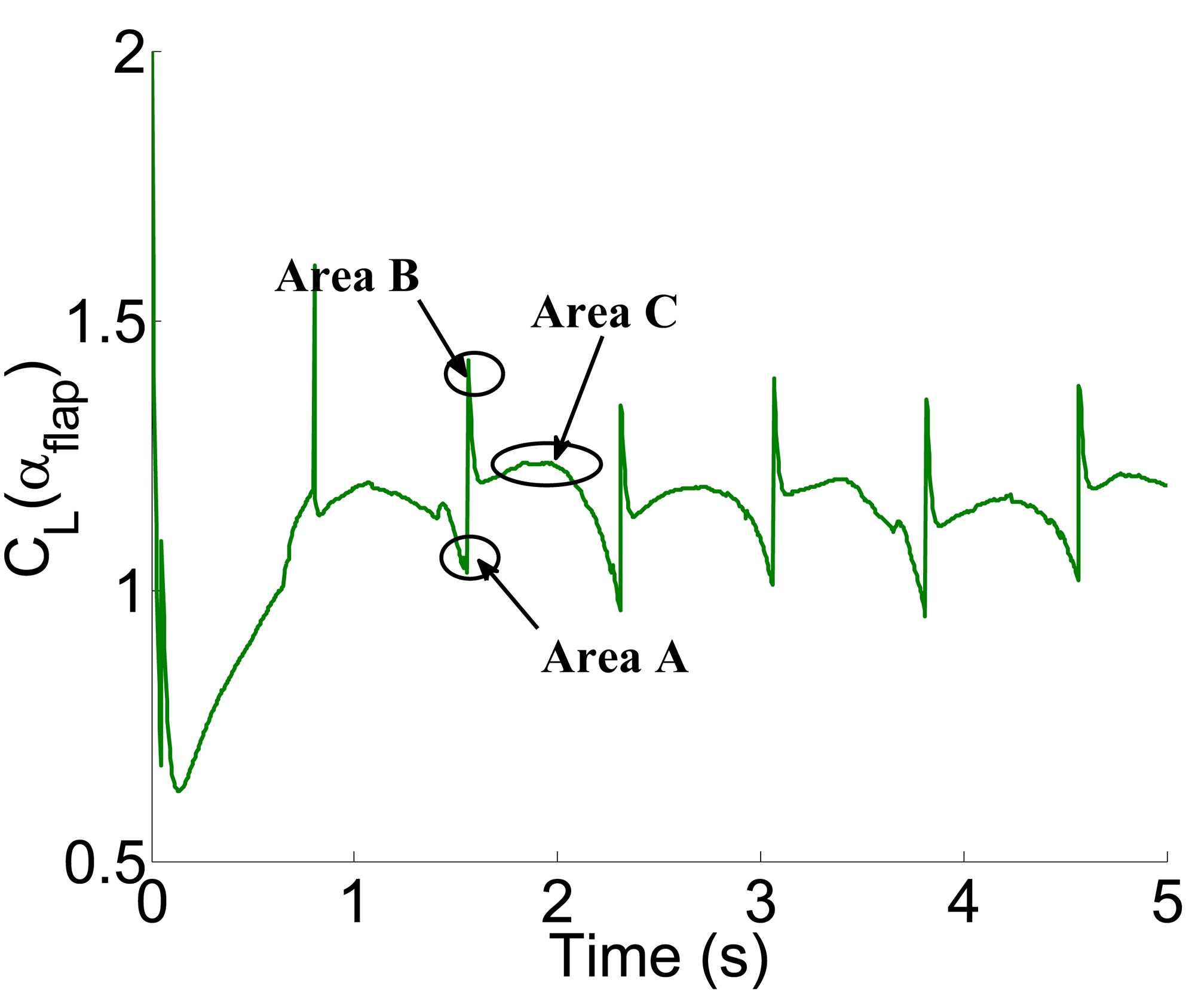

Supplement: S7 Fig — (TIF) [file pone.0214400.s007.tif]

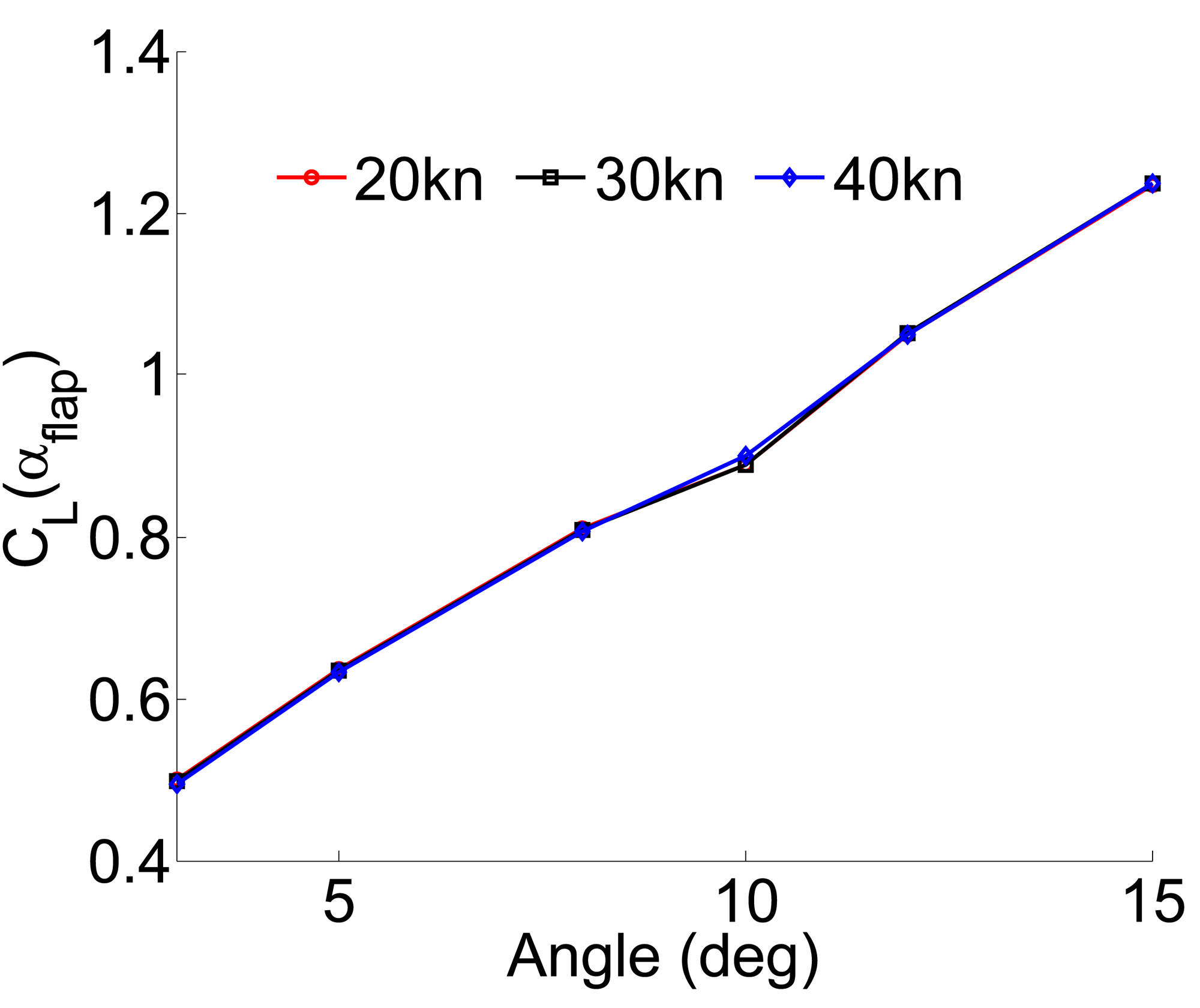

Supplement: S8 Fig — (TIF) [file pone.0214400.s008.tif]

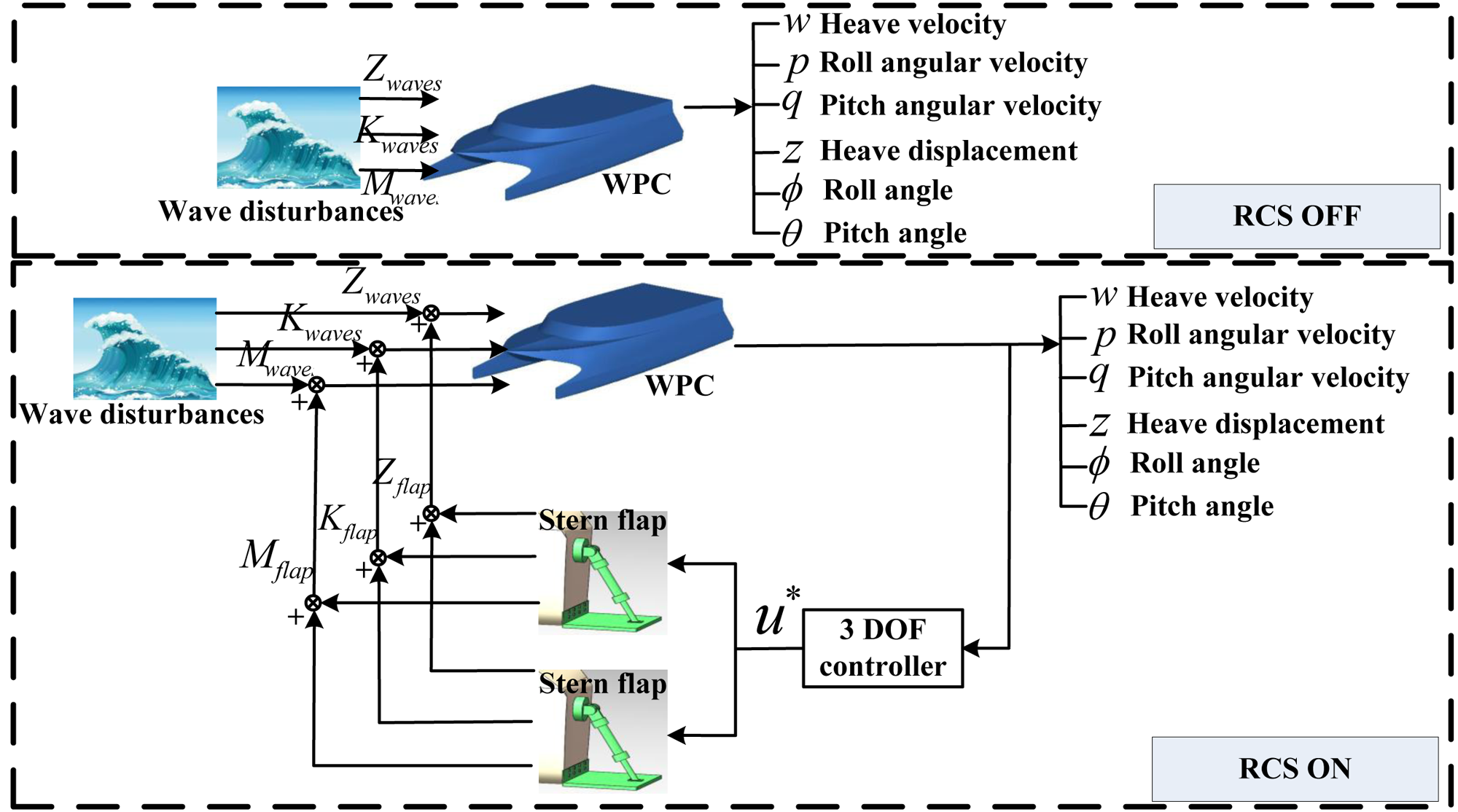

Supplement: S9 Fig — (TIF) [file pone.0214400.s009.tif]

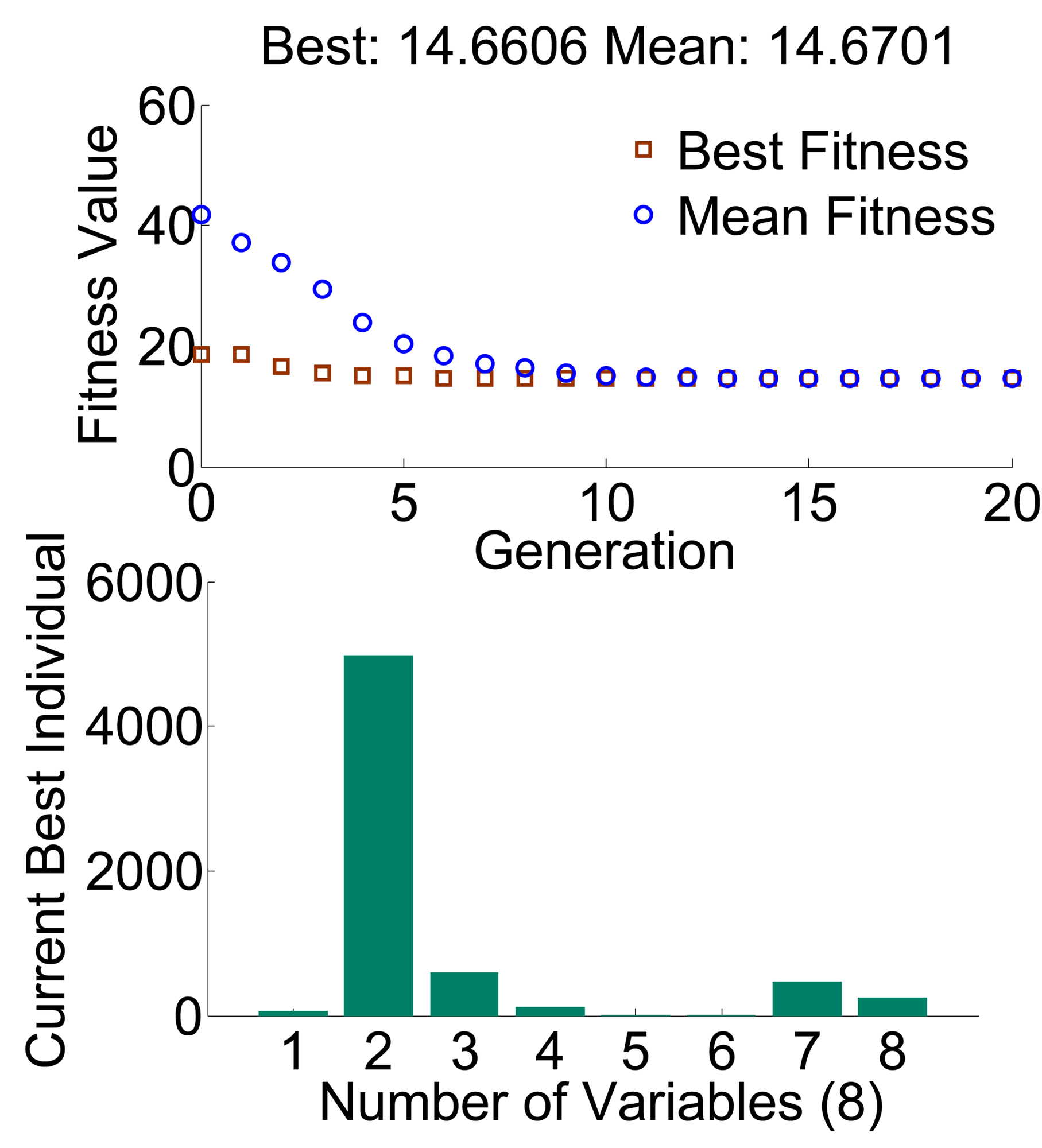

Supplement: S10 Fig — (TIF) [file pone.0214400.s010.tif]

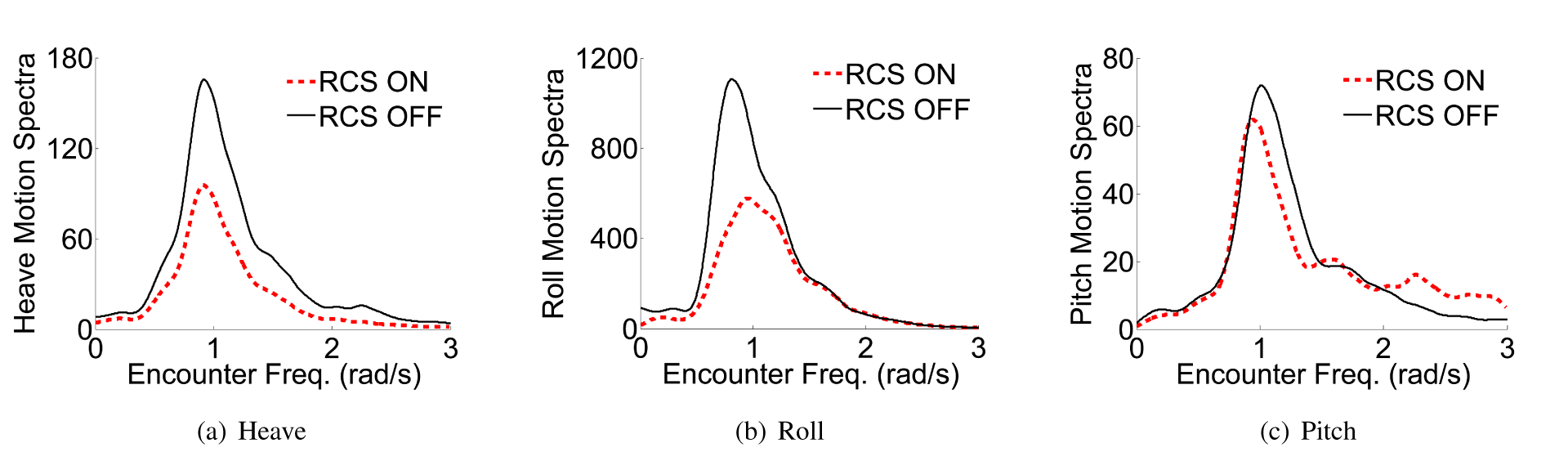

Supplement: S11 Fig — (TIF) [file pone.0214400.s011.tif]

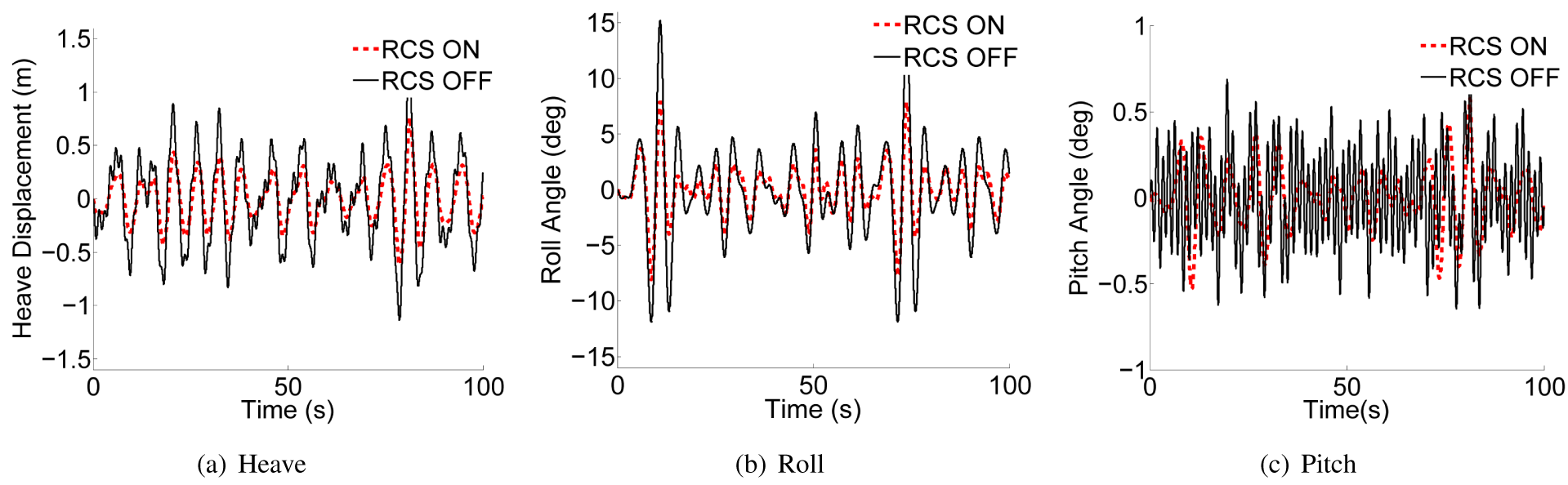

Supplement: S12 Fig — (TIF) [file pone.0214400.s012.tif]

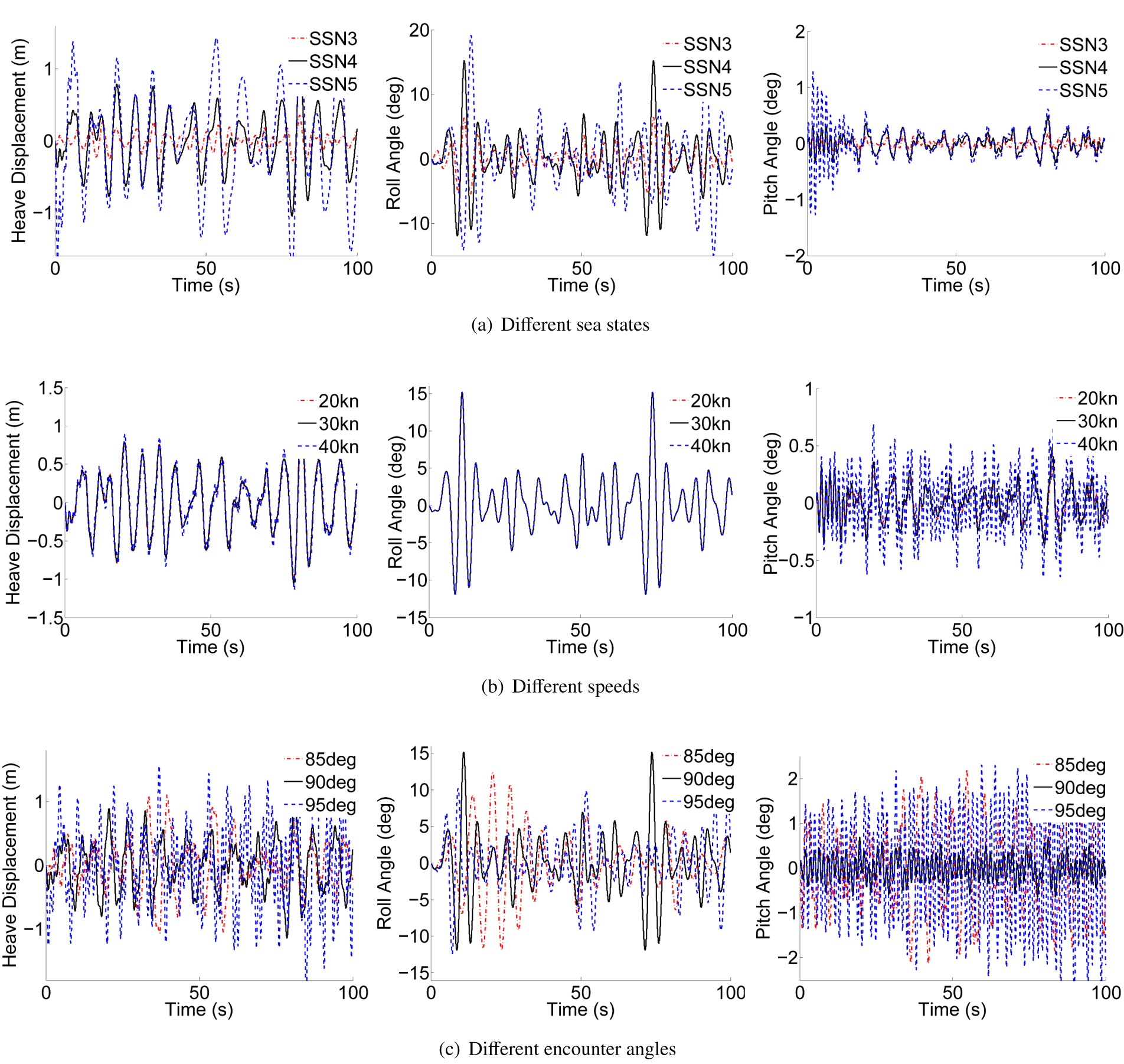

Supplement: S13 Fig — (TIF) [file pone.0214400.s013.tif]

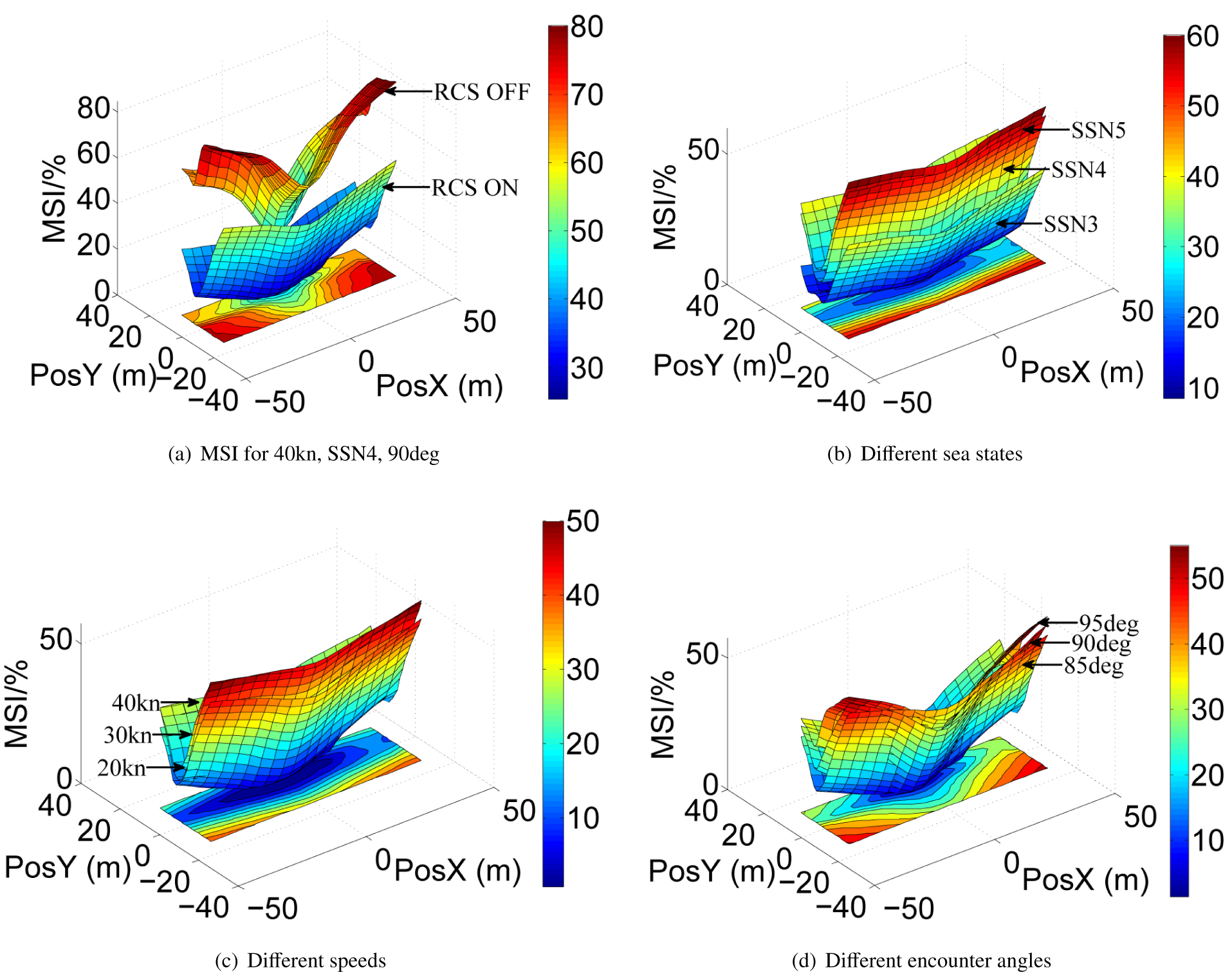

Supplement: S14 Fig — (TIF) [file pone.0214400.s014.tif]

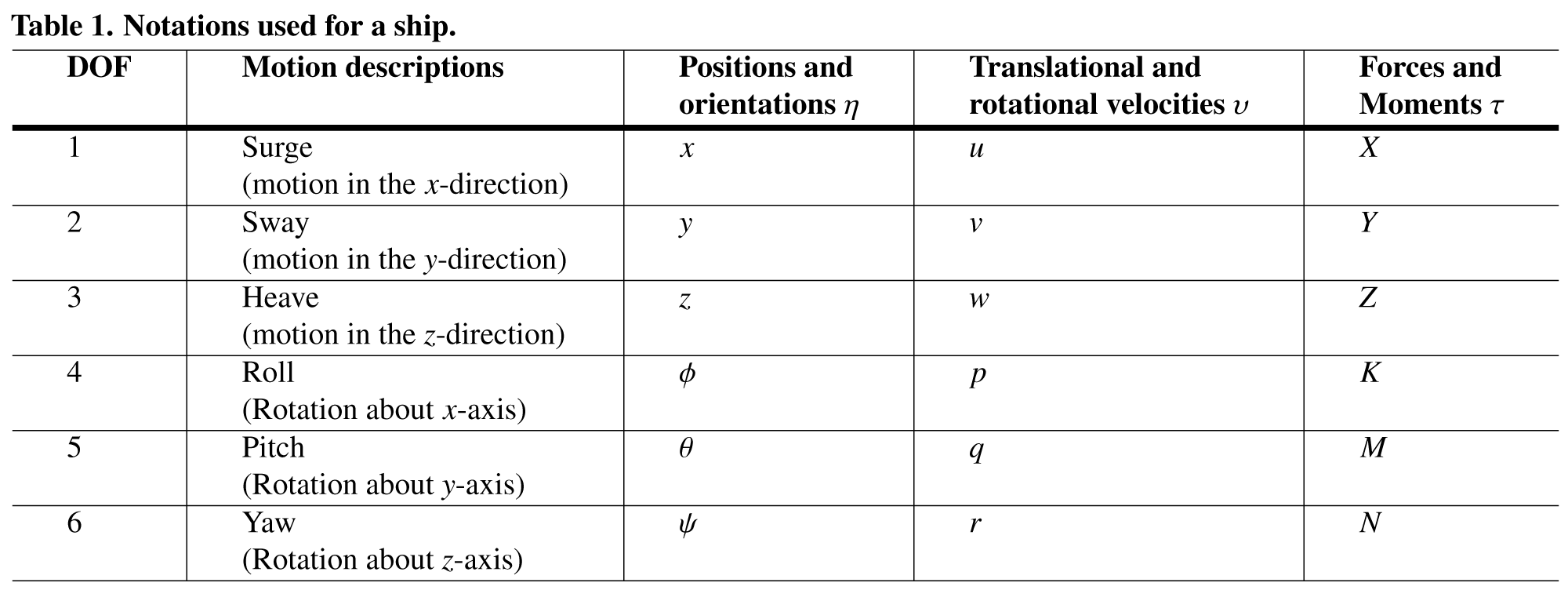

Supplement: S1 Table — (TIF) [file pone.0214400.s015.tif]

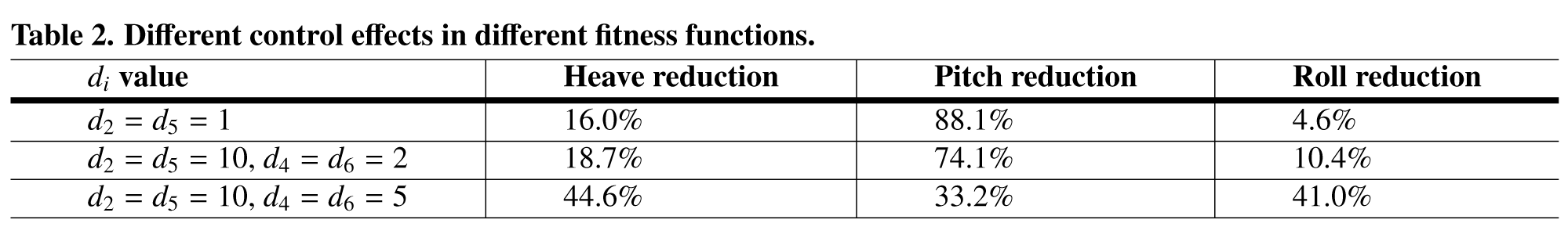

Supplement: S2 Table — (TIF) [file pone.0214400.s016.tif]

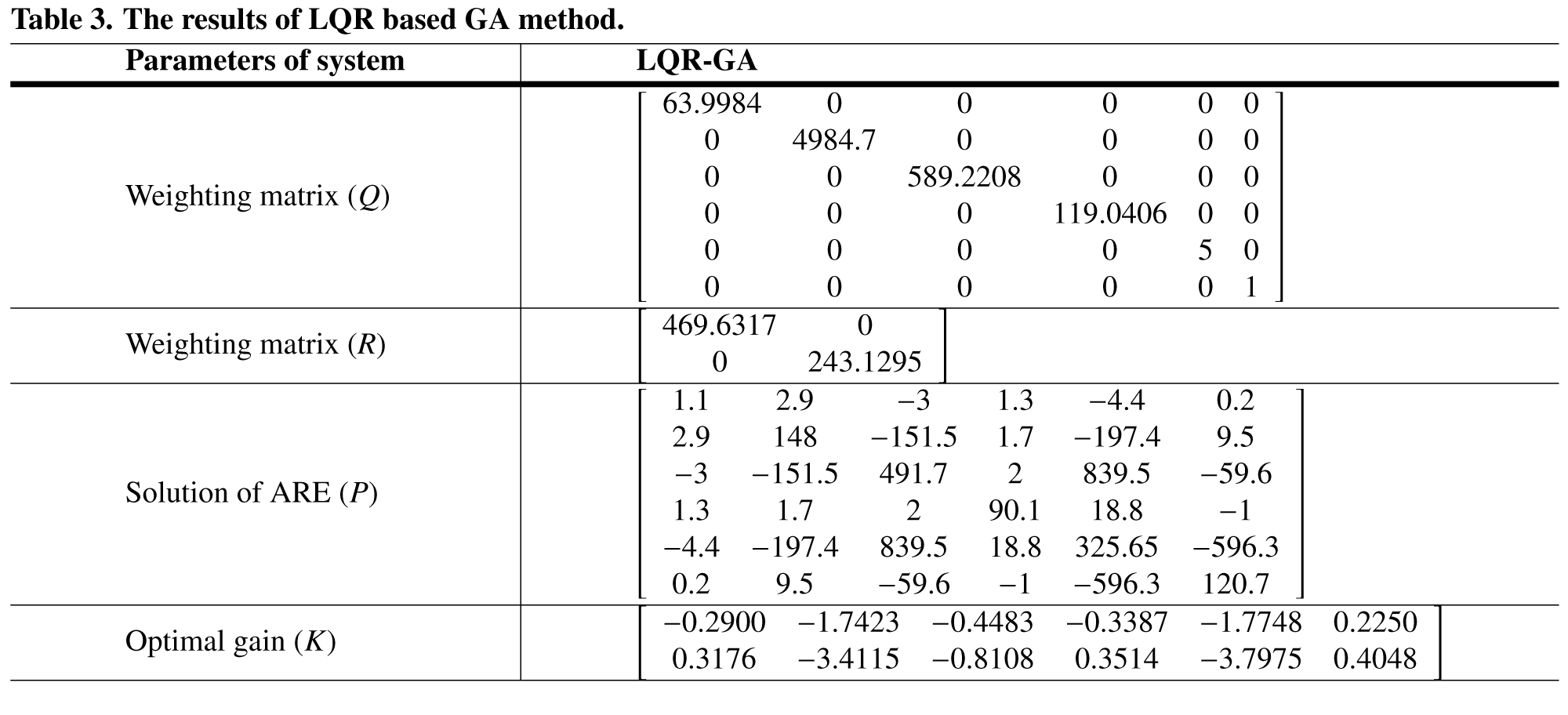

Supplement: S3 Table — (TIF) [file pone.0214400.s017.tif]

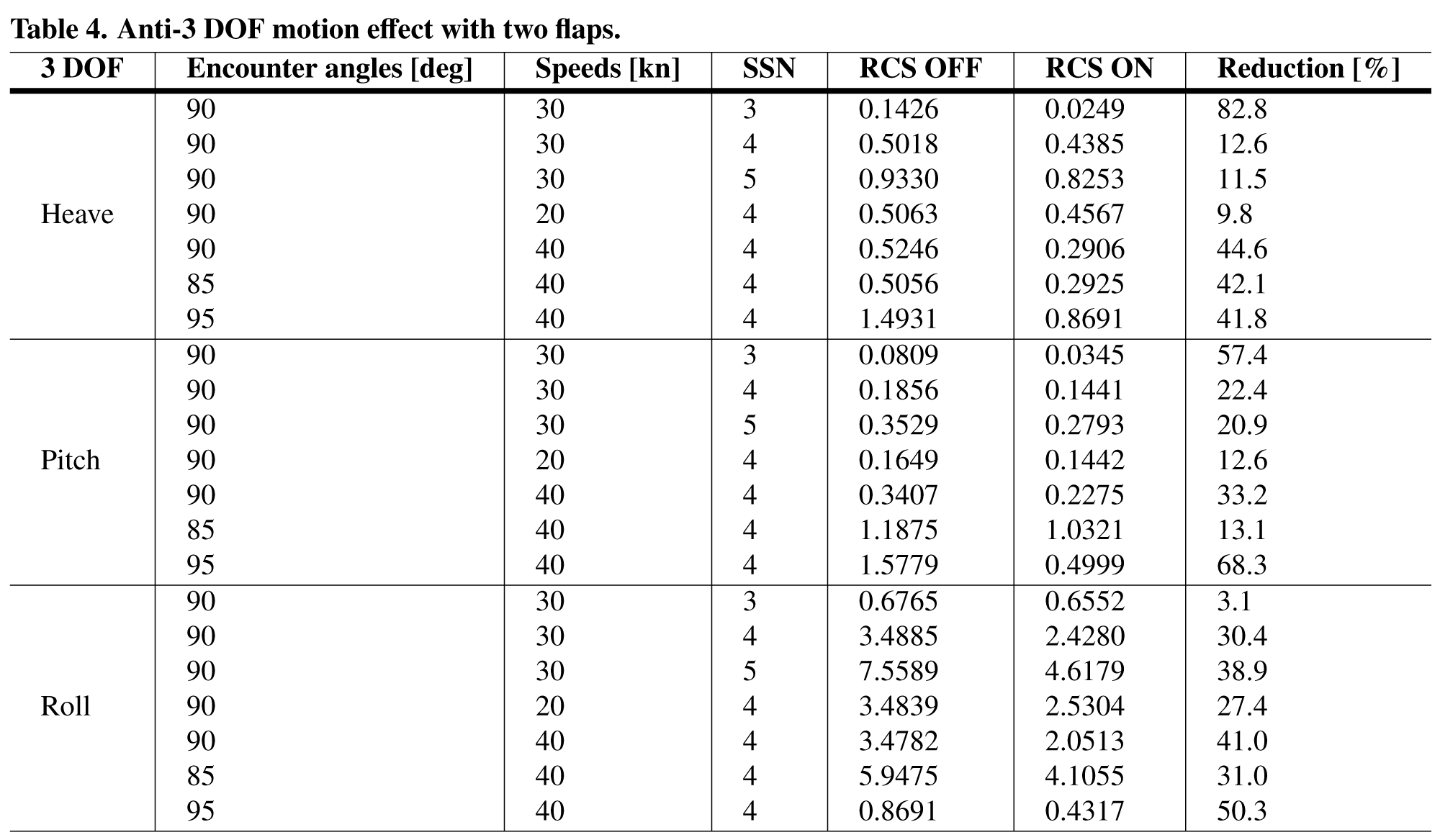

Supplement: S4 Table — (TIF) [file pone.0214400.s018.tif]
